# Understanding antidepressant change patterns in the UK Biobank

**DOI:** 10.1192/bjp.2026.10719

**Published:** 2026-07-17

**Authors:** Danyang Li, Chris Wai Hang Lo, Cathryn M. Lewis, Evangelos Vassos, Gerome Breen

**Affiliations:** Social, Genetic and Developmental Psychiatry Centre, King’s College London, London, UK; Department of Medical & Molecular Genetics, King’s College London, London, UK; UK National Institute for Health and Social Care Research (NIHR) Maudsley Biomedical Research Centre for Mental Health, South London and Maudsley Hospital, London, UK

**Keywords:** Antidepressant change, discontinuation, genetic factors, electronic health records

## Abstract

**Background:**

Antidepressants are the most frequently prescribed medications in psychiatry. Medical records of thousands of individuals provide a valuable opportunity to explore prescribing patterns and identify factors that influence treatment outcomes.

**Aims:**

To investigate antidepressant change patterns in depression and non-depression indications and assess clinical and genetic factors associated with outcomes of antidepressant treatment.

**Methods:**

Using primary care records from the UK Biobank, we examined outcomes including number of antidepressant changes and discontinuation due to either side-effects or inadequate response. Genetic analyses including heritability estimation, genetic correlation and polygenic score association were performed.

**Results:**

A total of 82 633 individuals were prescribed at least 1 antidepressant. Of these, 28 332 individuals with at least 1 primary care depression diagnosis were classified as the depression group, and 24 543 individuals without evidence of depression were classified as the non-depression group. Citalopram and fluoxetine were the most prescribed antidepressants for depression, whereas amitriptyline dominated prescriptions for non-depression indications. Individuals with depression were more likely to stay on antidepressants longer than those without depression and to follow preferred antidepressants that changed over time. Antidepressant changes and discontinuation were associated with a range of psychiatric and somatic conditions, including recurrent depression (early discontinuation: odds ratio = 1.96; late discontinuation: odds ratio = 2.63) and anxiety (early discontinuation: odds ratio = 1.37; late discontinuation: odds ratio = 1.99) in the depression group, and pain-related conditions in the non-depression group. Genetic analyses identified two novel variants associated with early discontinuation of selective serotonin reuptake inhibitors. Notable genetic overlap was shown between these outcomes and multiple psychiatric and physical traits, including the number of antidepressant changes with anxiety and depression (*r*_g_ = 0.81–0.83), and polygenic scores for depression and attention-deficit hyperactivity disorder showed significant predictive value with respect to treatment outcomes.

**Conclusions:**

These findings characterise antidepressant change patterns in primary care records and highlight the potential value of integrating clinical and genetic data to better understand factors associated with treatment outcomes.

Antidepressants are commonly prescribed for depression, general anxiety and a number of other indications, including neuropathic pain, sleep disorders and migraine.^1^ Despite the broad and widespread use of antidepressants, treatment often involves a lengthy trial-and-error process. Many individuals discontinue their prescriptions and undergo multiple switches among various antidepressants, largely owing to side-effects or inadequate response. Evidence suggests that only about one-third achieve remission after their first prescribed antidepressant,^2^ and 20–30% of individuals discontinue antidepressant treatment because of adverse effects.^3,4^ Understanding the factors that contribute to antidepressant discontinuation is essential to improving healthcare and outcomes for individuals receiving these medications.

In the UK, antidepressants are usually prescribed in primary care, and more than 30 different drugs across several classes have been widely used. Tricyclic antidepressants (TCAs) were among the earliest introduced and most commonly used for depression until the 1990s. Since then, selective serotonin reuptake inhibitors (SSRIs) have become the first-line choice for depression due to their lower mortality risk in cases of overdose.^5^ Some evidence suggests that discontinuation of treatment is associated with a history of prior discontinuation and a greater comorbidity burden.^3,6^ However, most of these findings come from short-term clinical cohorts or retrospective studies. In the long term, how real-world prescribing patterns and antidepressant choices in UK primary care patients have shifted over time and the longitudinal nature of treatment discontinuation remain poorly understood.

In addition to clinical indications, genetic factors can influence antidepressant outcomes. A recent genome-wide association study (GWAS) based on clinical trials reported that 13% of the variance in depression symptom remission during antidepressant treatment could be explained by common genetic variants.^2^ Other retrospective studies have shown that self-reported side-effects can be significantly predicted by polygenic scores (PGSs).^3,7^ Analyses of electronic health records (EHRs) provide a powerful alternative to clinical trials and retrospective cohort studies, given their use of comprehensive longitudinal data and substantially greater statistical power to identify clinical and genetic influences on antidepressant outcomes.^8,9^ Therefore, in this study, we used UK Biobank primary care records to investigate antidepressant prescribing patterns for both depression and non-depression indications. We aimed to describe the longitudinal trajectories of antidepressant changes, identify discontinuation patterns that were likely to be caused by side-effects or non-response, and evaluate clinical and genetic factors associated with these outcomes.

## Method

### Participants

The UK Biobank recruited approximately 500 000 participants aged 40–79 years between 2006 and 2010 through 22 assessment centres across the UK. Health and lifestyle information, as well as measures of hearing and cognitive function, were collected via touchscreen questionnaires and brief verbal interviews. Coded clinical events, such as diagnoses, clinical history, symptoms, prescriptions and administrative codes, were obtained through linkage with primary care records. All participants provided informed consent, demonstrating their understanding of the purpose, procedures, potential risks and benefits of the study before participating. Primary care prescription records were available for ∼230 000 participants. From these, a list of 37 prescribed antidepressants was curated according to a previous study^8^ (see Supplementary Table 1 and Supplementary Methods available at https://doi.org/10.1192/bjp.2026.10719).

The authors assert that all procedures contributing to this work comply with the ethical standards of the relevant national and institutional committees on human experimentation and with the Helsinki Declaration of 1975, as revised in 2013. All procedures involving human subjects were approved by the North West Multi-centre Research Ethics Committee as a Research Tissue Bank approval for UK Biobank research. Written informed consent was obtained from each participant in the UK Biobank before data collection.

### Antidepressant prescription for individuals with or without depression

In this study, individuals prescribed antidepressants with depression were defined as those who had at least one primary care diagnosis of depressive disorders and no diagnostic codes for psychotic disorders, bipolar disorder or substance use disorders.^8^ Prescriptions of amitriptyline and dosulepin were included only if the daily dosage met the therapeutic threshold for depression (≥50 mg for amitriptyline; ≥75 mg for dosulepin). Prescriptions occurring before the first recorded depression diagnosis were also excluded. Individuals prescribed antidepressants without depression were included if they had at least one antidepressant prescriptions but no depression measures in the UK Biobank (Supplementary Methods). We excluded multiple antidepressants that had been prescribed within 5 days to remove the effects of polytherapy. However, we acknowledge that this approach may not capture all augmentation strategies, particularly those in which additional medications were introduced sequentially outside this time window.

### Antidepressant change and discontinuation

Antidepressant change was defined as a switch from one antidepressant to another in the primary care records. We calculated the total numbers of different antidepressants prescribed across the overall sample and within the depression and non-depression groups. This measure served as a proxy for complex-to-treat status, representing cases in which the indication was not successfully treated. To distinguish discontinuation likely to be attributable to intolerable side-effects or non-response, we defined two discontinuation phenotypes as proxies for treatment cessation based on prescribing patterns among individuals with depression (Supplementary Fig. 1):

- Early discontinuation, likely due to side-effects: defined as cases in which an antidepressant was prescribed only once, followed by another antidepressant within 40 days, and the same antidepressant was not prescribed within 2 years before or after. This was intended to identify individuals who discontinued an antidepressant quickly, likely owing to intolerable side-effects, and the medication was subsequently avoided in primary care practice.
- Late discontinuation, likely due to non-response: defined as cases in which an antidepressant was prescribed for at least 42 days, with at least three prescriptions within a 6-month episode to ensure an adequate duration of treatment,^8^ and the drug was switched to another antidepressant within 40 days. This definition was intended to identify cases where the condition for which the medication was prescribed was not resolved and required further treatment.

### Hospital in-patient diagnoses of medical conditions

Primary and secondary diagnoses were retrieved from UK Biobank hospital in-patient records. We used three-digit ICD-10 codes, covering chapters I–XIV, chapter XVIII and chapter XXI of the ICD-10. For individuals with multiple records of the same diagnosis, only the first record was used. To extend the follow-up time to the period before the introduction of the ICD-10 in 1995, ICD-9 codes were mapped to the corresponding three-digit ICD-10 codes using general equivalence mappings from the Center for Disease Control (https://ftp.cdc.gov/pub/Health_Statistics/NCHS/Publications/ICD10CM/2018/).

### Statistical analyses

#### Antidepressant change pathways

To explore enriched antidepressant change pathways, we included sequential drug pairs prescribed to >10 individuals. To maximise statistical power, we focused on individuals initiating the most common antidepressants, which were citalopram and fluoxetine in the depression group and amitriptyline in the non-depression group. At the first prescription, enrichment of drug pairs was assessed by testing whether these individuals were more likely to switch to a specific second antidepressant compared with those starting on other drugs. Logistic regressions were applied, with the second antidepressant as the outcome and the first as the exposure, adjusting for gender, age at prescription, prescription year and data provider (England TPP, England Vision, Wales EMIS/Vision and Scotland EMIS/Vision; Supplementary Methods). For later stages, we first identified the directionality of each drug pair (drug A→drug B versus drug B→drug A) followed by more than half of individuals. At each antidepressant change, we tested whether drug A more frequently preceded drug B relative to other options, using logistic regression with drug B as the outcome and drug A as the exposure, adjusting for the covariates described above. Overlapping drug pairs were further combined into longitudinal trajectories of three or more prescriptions (e.g. drug A→drug B and drug B→drug C were combined into drug A→drug B→drug C; Supplementary Fig. 2). Associations were considered to be significant at false-discovery-rate-corrected *P* < 0.05 across all drug pairs within each initial antidepressant treatment.

#### Medical condition enrichment

For each antidepressant change outcome, we applied gamma regression to evaluate the enrichment of medical conditions among individuals with higher numbers of antidepressant changes. For each discontinuation outcome, logistic regression was used to estimate the risk of medical conditions in individuals who discontinued treatment compared with those who did not (Supplementary Methods). All models were adjusted for gender, data provider, participant birth year and year of first prescription. Only diagnoses occurring in more than 100 individuals per outcome were included. Diagnoses were considered to be significant at *P* < 0.05 after false discovery rate correction across all outcomes.

### Genetic analyses

#### Genome-wide association

GWAS were conducted using REGENIE v.3.1.3 (Regeneron Genetics Center, Tarrytown, New York; https://rgcgithub.github.io/regenie/) for five antidepressant outcomes, comprising the number of antidepressant changes in the full sample, depression and non-depression groups, as well as early and late discontinuation of SSRI treatment in the depression group, given predominant SSRI use in this population. GWAS were conducted with adjustment for batch, genotyping array, first ten genetic principal components, data provider, birth year and year of first prescription. For the X-chromosome, sex was included as an additional covariate. More details of genotyping, imputation and quality control can be found in the Supplementary Methods.

#### Heritability estimate, genetic correlation and PGS

Common genetic variant based heritability was estimated using both genome-wide complex trait analysis (GCTA) and linkage disequilibrium score regression (LDSC).^10,11^ Genetic correlations with selected psychiatric and physical traits were computed using LDSC. PGSs were calculated using SBayesR for major depressive disorder (MDD), schizophrenia, bipolar disorder, and attention-deficit hyperactivity disorder (ADHD).^12^ For antidepressant change outcomes, gamma regression was used to assess the explained variance of PGS associations. For early and late SSRI discontinuation, logistic regressions were applied, with Nagelkerke’s *R* ^2^ used to evaluate the explained variance. More details can be found in the Supplementary Methods.

## Results

### Characteristics of antidepressant prescription

A total of 82 633 UK Biobank participants had at least 1 recorded antidepressant prescription. The median age at first prescription was 54 years, and 65% of these participants were female. The median follow-up time was 9.6 years. Among these individuals, 28 332 had a primary care diagnosis of depression, whereas 24 543 had no evidence of depression identified in the UK Biobank. The remaining 29 758 individuals did not meet the criteria for either group, so they were excluded for the subgroup analyses. Compared with the non-depression group, individuals with a depression diagnosis were more likely to be female, to be younger at the time of the first prescription and to have a longer follow-up time (Supplementary Table 2).

**Fig. 1:**
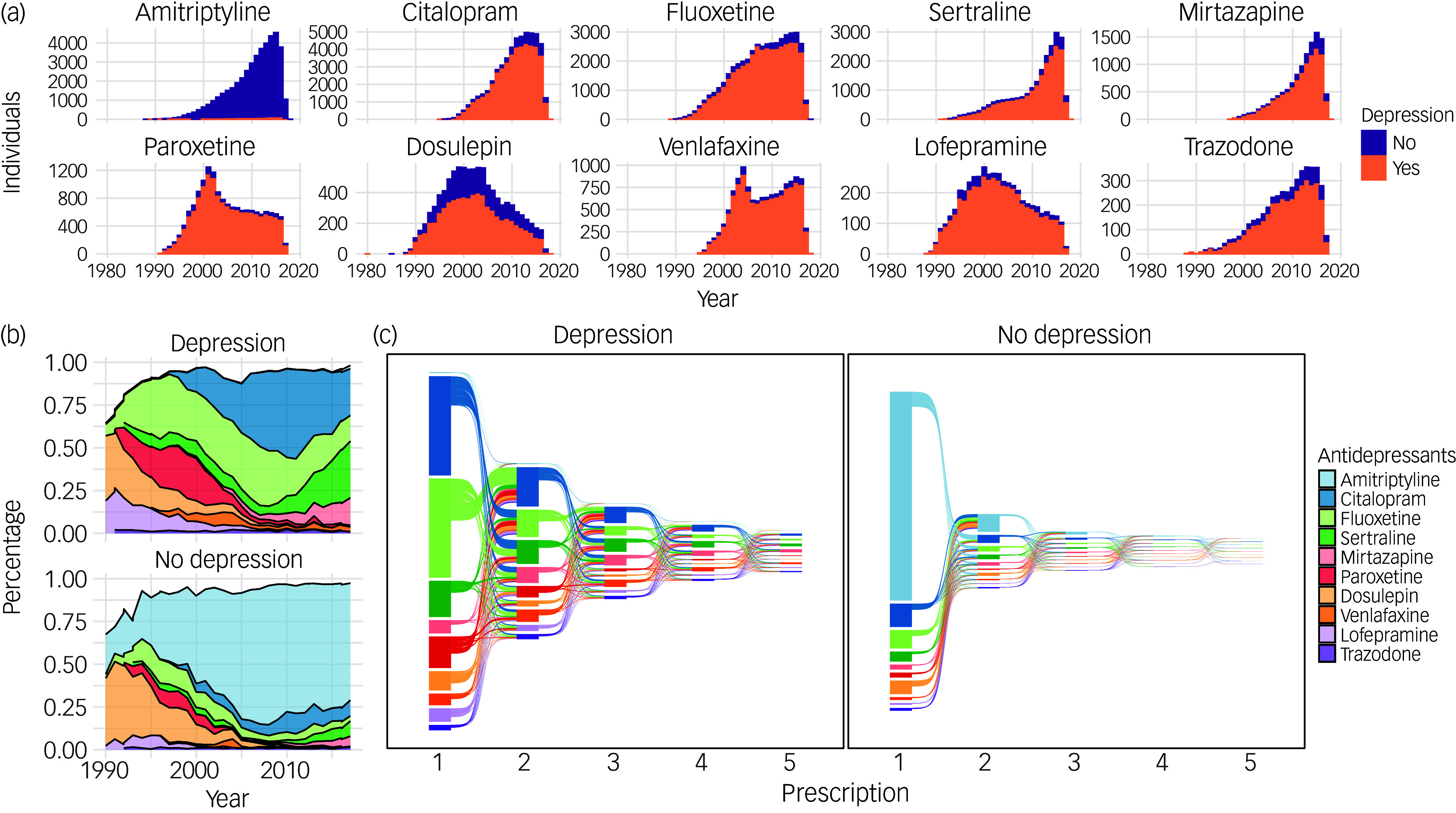
Fig. 1 long description. Most frequently prescribed antidepressants in the UK Biobank. (a) Number of individuals prescribed antidepressants across years, where orange indicates the number of individuals with depression, and purple shows the number of individuals without depression. (b) Percentages of the ten most commonly prescribed antidepressants from 1990 to 2018 at initial prescription. The blank space represents drugs not included among these ten antidepressants. (c) Trajectories of the ten most commonly prescribed antidepressants at the first five prescriptions. The *x*-axis shows the number of prescriptions, and the *y*-axis represents each individual with a prescribed antidepressant at each prescription. Each strip connects adjacent prescriptions of each individual, and blank spaces indicate that no antidepressant was prescribed.

In the depression group, the most commonly prescribed antidepressants were citalopram and fluoxetine, whereas amitriptyline was predominant in the non-depression group (Fig. 1(a), Supplementary Table 3). Around 1990, TCAs such as dosulepin and lofepramine were the most frequent first-line treatments in the depression group. Fluoxetine became dominant between 1990 and 2000, and citalopram took over in the early 2000s. In the past few years, sertraline has emerged as the most common first prescription (Fig. 1(b)). Among those without depression, amitriptyline (68%) consistently dominated first-line prescriptions across the years. Dosulepin was frequently prescribed in the early 1990s, but its use declined markedly over time. Most antidepressants showed comparable trends for subsequent prescriptions, although drugs including venlafaxine and mirtazapine showed increased proportions compared with those for initial treatment in the depression group (Supplementary Fig. 3). Numbers of individuals and percentages for the most frequently prescribed antidepressants stratified by data provider (England (Vision), England (TPP), Scotland, Wales) can be found in Supplementary Figs 4 and 5.

Numbers of antidepressant changes ranged from 1 to 13 in the depression group, and from 1 to 9 in the non-depression group (Supplementary Fig. 6). Antidepressant changes were more common among individuals with depression, with 47% receiving at least two different antidepressants, compared with 18% in the non-depression group (Fig. 1(c), Supplementary Fig. 6). Most individuals with depression started with SSRIs (78%), and 30% continuing SSRIs as the second antidepressant, followed by TCAs (5%) and serotonin–noradrenaline reuptake inhibitors (SNRIs; 4%) (Fig. 1(c), Supplementary Table 4). Augmentation with lithium consistently increased with the number of antidepressant changes in the depression group, from 0.94% at the first antidepressant prescription to 12.8% by the eighth (Supplementary Table 5).

### Antidepressant change temporal pathways

We focused on antidepressant change temporal pathways for the most commonly used initial antidepressants; these were citalopram and fluoxetine in the depression group, and amitriptyline in the non-depression group. Across these 3 initial treatments, we identified 100 drug pairs prescribed to more than 10 individuals (Supplementary Table 6). Among individuals who were first prescribed citalopram, the most frequent subsequent prescriptions were sertraline (27%), fluoxetine (24%), mirtazapine and escitalopram (Supplementary Fig. 7). For those starting with fluoxetine, the most likely second antidepressants were citalopram (37%), sertraline (16%), venlafaxine and paroxetine (Supplementary Fig. 8). In the non-depression group, individuals beginning with amitriptyline were more likely to switch to citalopram (22%), nortriptyline (16%), fluoxetine or duloxetine, and citalopram was commonly followed by mirtazapine or sertraline (Supplementary Fig. 9). All significant drug pairs stratified by year of prescription can be found in Supplementary Fig. 10.

### Antidepressant discontinuation likely due to side-effects or non-response in individuals with depression

We defined two types of antidepressant discontinuation: early discontinuation likely due to side-effects, and late discontinuation likely to be attributable to lack of response (Supplementary Fig. 1). In the depression group, 9% of individuals experienced at least one early discontinuation, whereas 12% had at least one late discontinuation. In each drug class, TCAs showed the highest rate of early discontinuation (7.3%), followed by SSRIs (5.9%) and SNRIs (5.2%) (*χ* ^2^ = 19.75, *P* = 5.14 × 10^−5^). By contrast, late discontinuation was more frequent among SSRIs (9.9%) compared with SNRIs (9.2%) and TCAs (8.8%) (*χ* ^2^ = 6.25, *P* = 0.044; Supplementary Table 7). Both discontinuation rates steadily increased across the first seven antidepressants (Supplementary Fig. 11) and showed differences across the SSRI, SNRI and TCA classes (Supplementary Fig. 12).

### Enrichment of medical conditions in antidepressant change and discontinuation

**Fig. 2:**
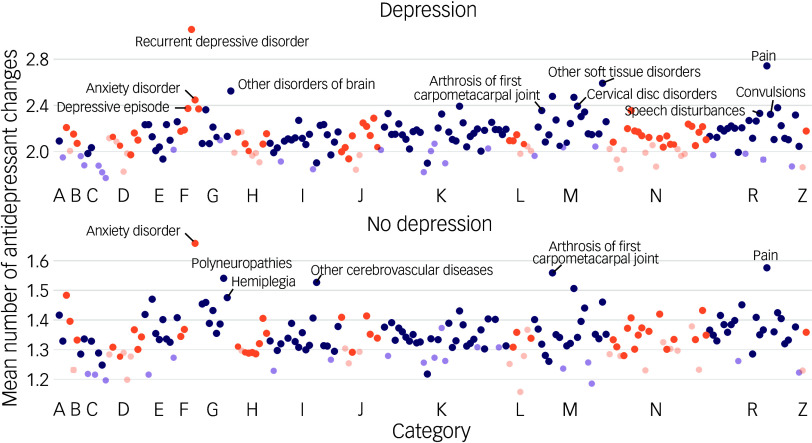
Mean numbers of antidepressant changes for each medical condition among individuals with and without depression. The *x*-axis shows the medical condition categories for ICD-10 codes A–N, R and Z. The *y*-axis shows the mean number of antidepressant changes for each medical condition in individuals with and without depression. Each dot represents a hospital in-patient diagnosis. Diagnoses within ICD-10 categories A, C, E, G, I, K, M and R are shown in purple, and those in categories B, D, F, H, J, L, N and Z are shown in orange. Dark colours indicate statistically significant diagnoses; light colours indicate non-significant ones. Effect sizes, 95% confidence intervals and sample sizes are listed in Supplementary Table 8.

From hospital in-patient diagnoses, we identified 207 medical conditions in the depression group and 187 in the non-depression group, for more than 100 individuals each. Of these, 158 and 146 conditions showed significant associations with antidepressant change in the depression and non-depression groups, respectively. In the depression group, frequent antidepressant change was most strongly enriched for recurrent depression, depressive episode, anxiety disorders, other brain disorders, pain syndromes and speech disturbances. The magnitude of these associations was modest, with effect size beta values in the range of 0.25–0.46. In the non-depression group, enrichment was observed for anxiety, pain, hemiplegia and polyneuropathies, with smaller effect sizes between 0.18 and 0.30 (Fig. 2, Supplementary Table 8). For SSRI discontinuation phenotypes, effect sizes were larger but remained within a moderate range. Early discontinuation was associated with 1.6–2.5-fold increased odds for some conditions, including recurrent depression, other brain disorders, chronic pain (R30, R52), and somatic conditions including cardiac arrhythmias, paroxysmal tachycardia and irritable bowel syndrome (IBS). Late discontinuation showed stronger associations, with odds ratios of around 2.0–2.6 for recurrent depression, depressive episode, anxiety, other brain disorders, speech disturbances, pain-related conditions (arthrosis of the first carpometacarpal joint [M18], pain [R52]), and somatic disorders such as obesity and respiratory failure (Supplementary Table 8).

### Single-nucleotide polymorphism (SNP)-based heritability

To further investigate genetic factors influencing antidepressant change and discontinuation, we performed GWAS for five antidepressant outcomes, including numbers of antidepressant changes in the full population, depression and non-depression groups, as well as SSRI early and late discontinuation in the depression group (Supplementary Table 9). Two variants showed genome-wide significance for SSRI early discontinuation in the depression group (rs184183447: beta = 1.29, *P* = 2.90 × 10^−10^, minor allele frequency = 0.011; rs142966989: beta = 1.29, *P* = 7.11 × 10^−10^, minor allele frequency = 0.011). These were located nearest to the *FLT1* gene. No significant variants were identified for the other phenotypes (Supplementary Figs 13–18). LDSC intercepts were approximately 1 in all these GWAS, suggesting no confounding factors (Supplementary Figs 13–17).

SNP-based heritability estimates for the number of antidepressant changes were comparable across groups, e.g. GCTA estimates were 2.6% (s.e. = 0.0053, *P* = 1.89 × 10^−7^), 2.5% (s.e. = 0.014, *P* = 0.034) and 2.8% (s.e. = 0.017, *P* = 0.040) in the full population, depression and non-depression groups, respectively. Discontinuation outcomes showed higher heritability, with GCTA estimates of 9.1% for early discontinuation and 4.6% for late discontinuation, although these did not reach statistical significance (early discontinuation: s.e. = 0.067, *P* = 0.084; late discontinuation: s.e. = 0.052, *P* = 0.19) (Supplementary Fig. 19). Genetic correlations between antidepressant outcomes can be found in Supplementary Table 10; none was significantly different from zero.

### Genetic correlation

**Fig. 3:**
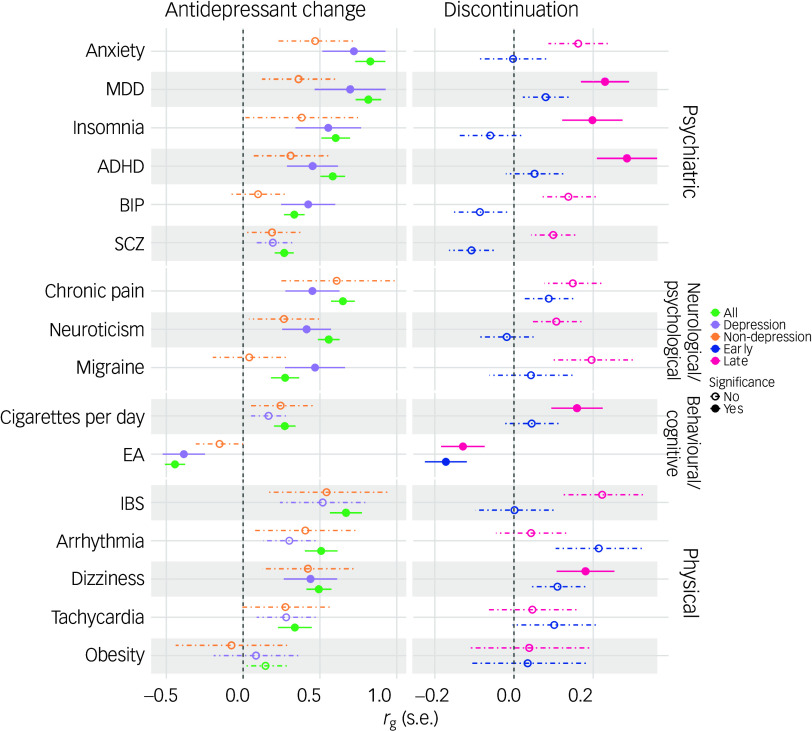
Genetic correlations of antidepressant change and discontinuation with psychiatric and physical traits. MDD, major depressive disorder; ADHD, attention-deficit hyperactivity disorder; BIP, bipolar disorder; SCZ, schizophrenia; EA, educational attainment; IBS, irritable bowel syndrome. Different colours represent antidepressant change and discontinuation outcomes. Solid lines and points represent significant correlations, whereas dashed lines and hollow dots show non-significant results after multiple testing correction.

We next tested the genetic correlation of antidepressant outcomes with various psychiatric and physical traits. The number of antidepressant changes in the full population was most strongly correlated with anxiety and MDD (*r*_g_ = 0.81–0.83; Fig. 3). Substantial genetic correlations were found with irritable bowel syndrome (IBS), chronic pain, insomnia, ADHD, neuroticism, arrhythmia and dizziness (*r*_g_ = 0.49–0.67). Moderate correlations were identified with tachycardia, bipolar disorder, migraine, smoking and schizophrenia (*r*_g_ = 0.27–0.33). Negative correlations were observed with educational attainment (*r*_g_ = −0.44). Antidepressant change in the depression group had stronger genetic correlations with most psychiatric and neurological traits, including anxiety, MDD, insomnia and migraine (*r*_g_ = 0.47–0.72), compared with the non-depression group. For discontinuation phenotypes, early discontinuation was significantly correlated with educational attainment (*r*_g_ = −0.17). Late discontinuation showed the highest correlation with ADHD (*r*_g_ = 0.29), followed by MDD (*r*_g_ = 0.23), insomnia (*r*_g_ = 0.20), dizziness (*r*_g_ = 0.18), smoking (*r*_g_ = 0.16) and educational attainment (*r*_g_ = −0.13).

### Polygenic scores

**Fig. 4:**
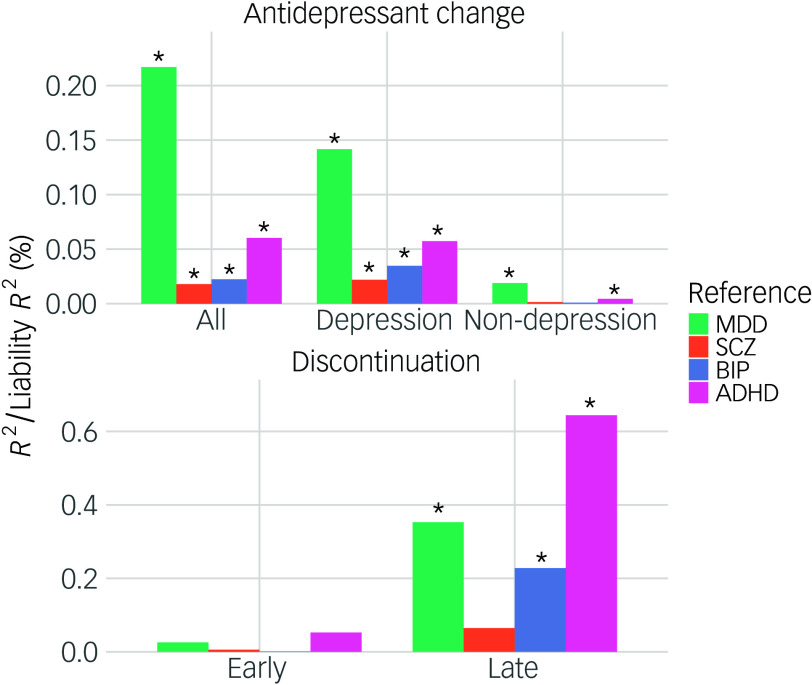
Explained variance of polygenic score for antidepressant change and discontinuation outcomes. MDD, major depressive disorder; SCZ, schizophrenia; BIP, bipolar disorder; ADHD, attention-deficit hyperactivity disorder. *Adjusted *P* < 0.05.

We calculated PGSs for MDD, schizophrenia, bipolar disorder and ADHD. The MDD PGS explained 0.22% (adjusted *P* = 1.05 × 10^−107^) of the variance in the number of antidepressant changes in the full population, 0.14% (adjusted *P* = 2.50 × 10^−22^) of that in the depression group, and 0.02% (adjusted *P* = 4.30 × 10^−6^) of that in the non-depression group (Fig. 4). Significant associations were also observed for the ADHD PGSs across all three groups, and for the bipolar disorder and schizophrenia PGSs in the full population and depression group (Fig. 4, Supplementary Table 11). For SSRI early discontinuation, the MDD PGS explained 0.03% (adjusted *P* = 0.49) of the variance, and the ADHD PGS accounted for 0.05% (adjusted *P* = 0.34). For SSRI late discontinuation, PGS for ADHD showed the strongest association with the explained variance of 0.64% (adjusted *P* = 5.07 × 10^−7^), followed by PGS for MDD (liability *r* ^2^ = 0.35%, adjusted *P* = 2.33 × 10^−5^) and bipolar disorder (liability *r* ^2^ = 0.23%, adjusted *P* = 0.0036).

## Discussion

Our study is the first to characterise the longitudinal landscape of antidepressant change and discontinuation patterns for both depression and non-depression indications in the UK Biobank. Using primary care records, we found that individuals with depression were most frequently prescribed SSRIs such as fluoxetine, citalopram and sertraline, whereas TCAs, particularly amitriptyline, were most frequently prescribed among those without depression. Over the past 30 years, preferred antidepressant choices have shifted markedly, reflecting evolving clinical recommendations in UK primary care. We also identified two discontinuation phenotypes likely to reflect intolerable side-effects or inadequate response. All these phenotypes were enriched for a range of psychiatric and somatic conditions. Genetic analyses revealed substantial overlap of antidepressant change and discontinuations with multiple psychiatric and physical traits, and PGSs could explain a notable proportion of outcome variance.

We observed distinct patterns of antidepressant prescribing between the depression and non-depression groups. Consistent with previous reports, SSRI prescriptions for depression, including fluoxetine and citalopram, increased rapidly from the early 1990s, followed by a marked increase in prescriptions of sertraline after 2009, due to its favourable balance between efficacy and tolerability.^13–15^ Among TCAs, prescription of dosulepin and lofepramine declined steadily after 1990, whereas that of low-dose amitriptyline increased substantially, covering a range of indications including chronic pain, sleep disorders, anxiety, migraine, psychological distress and IBS.^13,14,16^ We found broadly consistent antidepressant prescribing trends across the four primary care data providers from England, Scotland and Wales in the UK Biobank. As in healthcare systems in countries outside the UK,^17–19^ antidepressant use has increased steadily over time, and SSRIs remain the predominant treatment for depression.

We observed that individuals with depression were more likely to switch to another antidepressant and to follow longer switching pathways within antidepressant treatments compared with those without depression. As antidepressants represent the primary pharmacological treatment for depression, individuals with depression have greater exposure to multiple antidepressant options over time, increasing the likelihood of observed switching within this drug class. For non-depression indications such as pain or IBS, treatment changes may involve switching to non-antidepressant medications (e.g. analgesics or corticosteroids), which were not captured in this study. Despite this, the switching pathways, particularly in those with depression, were complex and varied over time, partly reflecting changes in UK primary care practice. For example, individuals starting on fluoxetine could switch to other SSRIs, such as citalopram or sertraline, or to TCAs such as lofepramine and dosulepin.^20^ However, switches to TCAs mainly occurred before the early 2000s, with changing to SSRIs preferred in later years, in line with updated UK clinical guidelines recommending switching to an alternative SSRI as a first step before considering antidepressants with different mechanisms or augmentation strategies.^15^ Subsequent transitions to other drug classes, such as SNRIs or TCAs, or increasing use of augmentation such as lithium followed with the increased number of antidepressant changes likely to represent treatment escalation in more complex or persistent cases. TCAs showed the highest rates of early discontinuation, consistent with their known tolerability issues.^3,21^ By contrast, late discontinuation rates were initially similar across drug classes but diverged after the third antidepressant, with SSRIs showing higher rates; this may have corresponded to individuals not responding well to serotoninergic antidepressants, leading to treatment choices involving more diverse neurotransmitters or mechanisms. As primary care physicians may consider switching to TCAs or other classes after non-response to SSRIs, and our definition only included antidepressant monotherapy switches excluding drug augmentation and other treatment strategies such as electroconvulsive therapy, these results need to be interpreted with caution.

Further analyses of hospital in-patient diagnoses showed that frequent antidepressant changes were associated with anxiety, pain conditions, and diagnoses of somatic conditions that can have pain-related symptoms, such as migraine and osteoarthritis. In the depression group, additional associations included recurrent depression, speech disturbance and brain disorders (e.g. encephalopathy). In addition to psychiatric disorders, cardiac disorders such as arrhythmias and gastrointestinal issues including IBS were linked to early discontinuation. These diagnoses have commonly been reported as concerning side-effects of SSRIs.^22,23^ For example, high doses of citalopram have been associated with prolongation of the QTc interval, possibly leading to more frequent cardiac monitoring and prompt changes of medication in cases of arrhythmias. Late discontinuation was associated with obesity, in line with previous studies showing that higher body mass index reduced effectiveness and increased risk of treatment resistance.^24^ Co-prescription of non-steroidal anti-inflammatory drugs (NSAIDs) with SSRIs was also associated with increased risk of adverse effects, including gastrointestinal bleeding and brain haemorrhage.^25,26^ As cytochrome P450 (CYP)2D6 is a key metabolic enzyme for both opioids and antidepressants, concomitant use of CYP2D6-metabolised opioids with antidepressants that inhibit the CYP2D6 enzyme may reduce analgesic effects, potentially worsening pain symptoms and increasing the risk of impaired physical functioning and mental health problems, including depression.^27^ These results could further worsen antidepressant outcomes, leading to treatment switching.

The gene nearest to the variants associated with SSRI early discontinuation (rs184183447 chr13:28515228, rs142966989 chr13:28496354), was *FLT1* (chr13:28300346-28495128), encoding vascular endothelial growth factor receptor 1. However, neither variant showed a significant effect in the independent Genetic Links to Anxiety and Depression study for the self-reported outcome of discontinuation due to side-effects (Supplementary Table 12, Supplementary Methods). We acknowledge the limited power of the study with respect to discontinuation outcomes and the possibility that the low minor allele frequencies (∼1%) of these two variants might have increased the risk of false positives. A more stringent quality threshold could improve the robustness of the findings, especially for the analysis in smaller subsamples of the UK Biobank, although some possible significant findings could be filtered out. In addition, SNP-based heritability, especially for early discontinuation of SSRIs, reached 9%, exceeding the earlier value of 4% in a study capturing SSRI switching from primary care records.^20^ However, the heritability for discontinuation phenotypes was not significantly different from zero, probably owing to the limited sample size. As additional biobanks and linked prescription datasets become available, future meta-analyses across multiple cohorts will be essential to achieve adequate power to detect reproducible findings.^28,29^

Genetic correlations showed shared genetic predisposition between antidepressant change and a range of psychiatric disorders, including MDD, neuroticism and ADHD, consistent with previous findings for treatment-resistant depression.^8^ As expected, the depression group showed higher correlations with most psychiatric and neurological traits, including anxiety, MDD, neuroticism, insomnia, bipolar disorder and migraine, whereas the non-depression group tended to have stronger correlations with chronic pain and physical conditions including IBS. For the discontinuation phenotypes, although most traits were not significantly correlated with early discontinuation, we observed the highest genetic correlation between early discontinuation and arrhythmia, suggesting a possible shared genetic liability to traits related to specific side-effects, especially cardiac symptoms.

We showed significant associations of PGSs for psychiatric traits with the number of antidepressant changes and SSRI late discontinuation; these results suggest an overlap of genetic architecture between psychiatric disorders and antidepressant outcomes and highlight the potential of polygenic approaches to stratify patients at a population level. However, the effect sizes were generally modest, with less than 1% of variance explained for antidepressant outcomes, limiting the clinical utility of the findings for individual-level prediction. As in prior studies of self-reported non-response and treatment-resistant depression,^8,30^ we identified a significant genetic correlation and PGS association between late discontinuation and ADHD. Notably, it has been reported that fewer than 20% of adult ADHD cases are detected in primary care,^31^ and studies show that ADHD could increase the risk of antidepressant treatment resistance.^32,33^ One possible explanation is emotional dysregulation, which has been reported to be worse when ADHD co-occurs with major depression and to further increase the risk of treatment resistance.^33^ These findings underscore the importance of effective and reliable ADHD assessment, particularly for individuals showing poor antidepressant response.

Our study had some limitations. The primary care EHR data covered only about half of UK Biobank participants, limiting overlap with other subsets of samples. Available prescription records were primarily from the 1990s onward, so treatment histories for older antidepressants such as TCAs were incomplete. Although there was no information on clinician behaviour, nor on clinical measures such as depression symptoms and severity before and after treatment, and most side-effects were likely to have been underreported in the structured EHR data,^34^ the early and late discontinuation we measured using treatment continuation and antidepressant switching has been reported in treatment-resistant depression,^8^ and our genetic findings showed good concordance with self-reported outcomes.^30^ However, our relatively stringent definitions of discontinuation captured only a subset of individuals with clear patterns of likely experiencing side-effects or non-response. As a result, these definitions may underestimate the true rate of antidepressant discontinuation in clinical practice. The number of antidepressant changes, on the other hand, is a heterogeneous measure that may reflect multiple clinical processes, including lack of response, side-effects or guideline-driven prescription changes. This measure can serve as a proxy for overall treatment complexity, supporting its utility in evaluating complex treatment trajectories in primary care records, but we acknowledge its limited representation of mechanism specificity. As this was an observational study using routine primary care records, the findings are hypothesis-generating and do not establish causal relationships. Antidepressant dosage and duration are important determinants of treatment outcomes. Subtherapeutic dosing and shorter treatment duration are associated with poor response, whereas adequate therapeutic dosing improves remission but may also increase the risk of side-effects.^35,36^ Further work is needed to determine how dosage and duration relate to the antidepressant outcomes defined in medical records. Last, different ethnic groups often have distinct antidepressant treatment patterns due to various social and cultural factors that influence their access to mental healthcare and adherence to treatment.^37,38^ Given that UK Biobank participants are primarily of European ancestry, further studies using primary care records with diverse population ancestries are required.

In summary, our study is the first to investigate longitudinal antidepressant changes using primary care records from the UK Biobank. We identified distinct antidepressant prescribing patterns between depression and non-depression indications, reflecting differences in treatment goals. We provide a pragmatic approach to assess treatment complexity and discontinuation likely due to side-effects or non-response, particularly among individuals with depression. Discontinuation rates differed across antidepressant classes and increased alongside treatment switching. Antidepressant outcomes were associated with a range of comorbid conditions, which may increase risk of side-effects or non-response, highlighting a potential need for closer monitoring and earlier consideration of treatment tolerability or alternative strategies. Although significant genetic differences were observed across antidepressant outcomes, the predictive utility of these findings at the individual level remained limited. Overall, our study provides a valuable framework for assessing antidepressant prescribing patterns in EHR data and highlights the potential of integrating clinical and genetic information to better understand factors influencing treatment outcomes, ultimately supporting more personalised approaches to antidepressant treatment.

## Supporting information

10.1192/bjp.2026.10719.sm001 Li et al. supplementary material 1 Li et al. supplementary material

10.1192/bjp.2026.10719.sm002 Li et al. supplementary material 2 Li et al. supplementary material

## Data Availability

UK Biobank data are publicly available to bona fide researchers upon application at http://www.ukbiobank.ac.uk/using-the-resource/. All software and analytical methods used in this study are publicly available. Genetic analysis code is accessible at https://github.com/DanyangLi107/AD_Change_Pattern_UKB.

## Supplementary material

The supplementary material is available online at https://doi.org/10.1192/bjp.2026.10719

## Fig. 1 Long description

Panel A: This panel contains multiple line graphs showing the number of individuals prescribed various antidepressants across years. The antidepressants include Amitriptyline, Citalopram, Fluoxetine, Sertraline, Mirtazapine, Paroxetine, Dosulepin, Venlafaxine, Lofepramine, and Trazodone. The x-axis represents the year ranging from 1980 to 2020, and the y-axis represents the number of individuals. The orange color indicates individuals with depression, while the purple color indicates individuals without depression. Panel B: This panel contains two line graphs showing the percentages of the ten most commonly prescribed antidepressants from 1990 to 2018 at initial prescription. The top graph represents individuals with depression, and the bottom graph represents individuals without depression. The x-axis represents the year, and the y-axis represents the percentage. Different colors represent different antidepressants. The blank space represents drugs not included among these ten antidepressants. Panel C: This panel contains two Sankey diagrams showing the trajectories of the ten most commonly prescribed antidepressants at the first five prescriptions. The left diagram represents individuals with depression, and the right diagram represents individuals without depression. The x-axis shows the number of prescriptions, and the y-axis represents each individual with a prescribed antidepressant at each prescription. Each strip connects adjacent prescriptions of each individual, and blank spaces indicate that no antidepressant was prescribed.

Navigate back to Fig. 1.
