# Supplementary material for: Understanding antidepressant change patterns in the UK Biobank
Source: Br J Psychiatry. 2026 Jul 17:1–9. Online ahead of print. doi: 10.1192/bjp.2026.10719 (PMC13384193; doi:10.1192/bjp.2026.10719)
Supplement: Li et al. supplementary material 1 — Li et al. supplementary material [file S0007125026107193sup001.pdf]

Supplementary

Supplementary Figures *3*

Supplementary Figure 1. Definition of discontinuation phenotypes 3

Supplementary Figure 2. Overview of antidepressant change pathway identification in the UK Biobank 4

Supplementary Figure 3. Proportion of the most ten common antidepressants prescribed at the second and later prescriptions 5

Supplementary Figure 4. Number of individuals prescribed most common ten antidepressants in each data provider (England TTP, England Vision, Scotland, Wales) across years 6

Supplementary Figure 5. Percentage of the most common ten antidepressants at the initial prescription from 1996 to 2018 across data providers 7

Supplementary Figure 6. Total number of antidepressant changes in depression and non-depression groups 8

Supplementary Figure 7. Antidepressant change pathways starting from citalopram in the depression group 9

Supplementary Figure 8. Antidepressant change pathways starting from fluoxetine in the depression group 10

Supplementary Figure 9. Antidepressant change pathways starting from amitriptyline in the non-depression group 11

Supplementary Figure 10. Percentage of significant drug pairs changed across years 12

Supplementary Figure 11. Early and late discontinuation rates at the first ten prescribed antidepressants 13

Supplementary Figure 12. Early and late discontinuation rates of each antidepressant class across number of different antidepressants prescribed 14

Supplementary Figure 13. GWAS association of the number of antidepressant changes in all population 15

Supplementary Figure 14. GWAS association of the number of antidepressant changes in the depression group 16

Supplementary Figure 15. GWAS association of the number of antidepressant changes in the non-depression group 17

Supplementary Figure 16. GWAS association of SSRI early discontinuation 18

Supplementary Figure 17. GWAS association of SSRI late discontinuation 19

Supplementary Figure 18. Regional plot of SSRI early discontinuation 20

Supplementary Figure 19. SNP heritability (SNP-h2) estimated by GCTA and LDSC 21

Supplementary Tables *22*

Supplementary Table 1. Prescribed antidepressants in the UK Biobank 22

Supplementary Table 2. Characteristics of individuals with antidepressant prescription in depression and non-depression groups. 23
Supplementary Table 3. The top ten most prescribed antidepressants in the UK Biobank 24
Supplementary Table 4. The top ten most prescribed antidepressants at the first five prescriptions 25
Supplementary Table 5. Percentage of co-prescription between lithium and antidepressants 26
Supplementary Table 7. Early and late discontinuation rates across antidepressant classes in individuals with depression 27
Supplementary Table 9. GWAS sample size of number of antidepressant changes and discontinuation outcomes 28
Supplementary Table 10. Genetic correlation among antidepressant changes and discontinuation phenotypes 29
Supplementary Table 12. Chi-squared tests of discontinuation outcomes with two genetic variants in the GLAD and UK Biobank 30
Supplementary Methods 31

Supplementary Figures

Supplementary Figure 1. Definition of discontinuation phenotypes

Early discontinuation likely due to intolerable side effects:

1. An antidepressant prescribed only once followed by another antidepressant within 40 days
2. This antidepressant is not prescribed before or after within 2 years

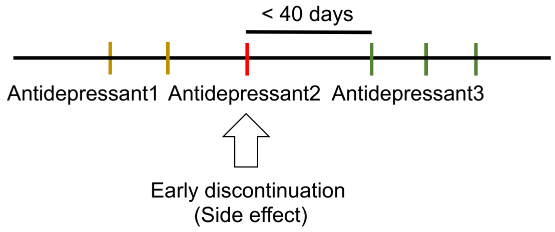

Late discontinuation likely due to nonresponse:

1. An antidepressant prescribed at least 6 weeks in a prescription episode
2. This antidepressant was prescribed more than 3 times in this episode
3. This drug was switched to another antidepressant within 40 days

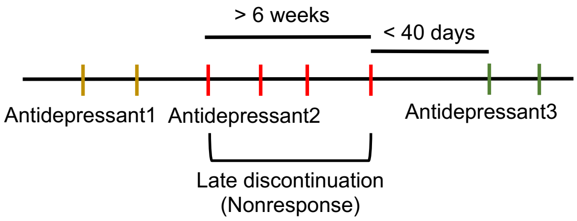

Supplementary Figure 2. Overview of antidepressant change pathway identification in the UK Biobank

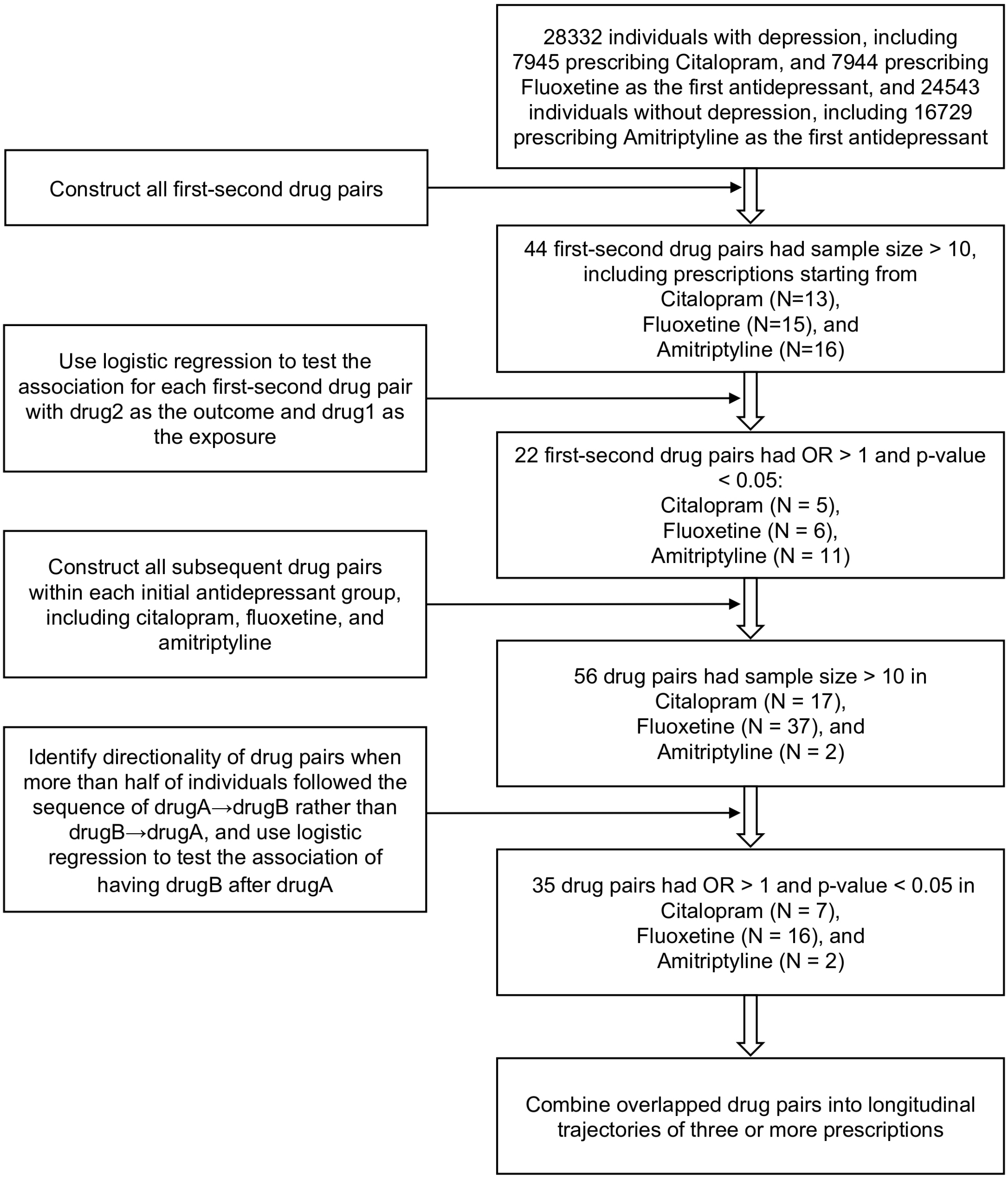

Supplementary Figure 3. Proportion of the most ten common antidepressants prescribed at the second and later prescriptions

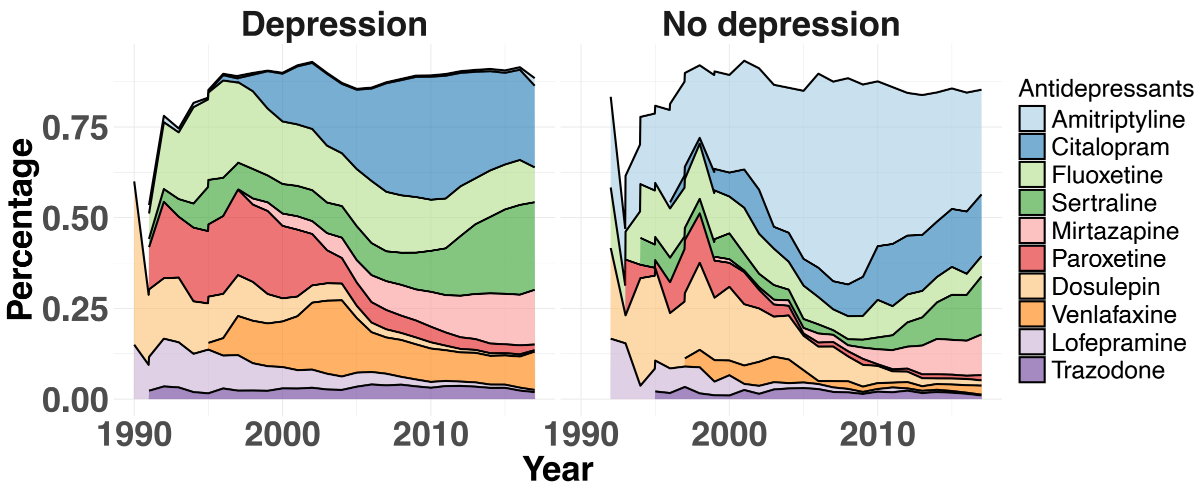

Supplementary Figure 4. Number of individuals prescribed most common ten antidepressants in each data provider (England TTP, England Vision, Scotland, Wales) across years

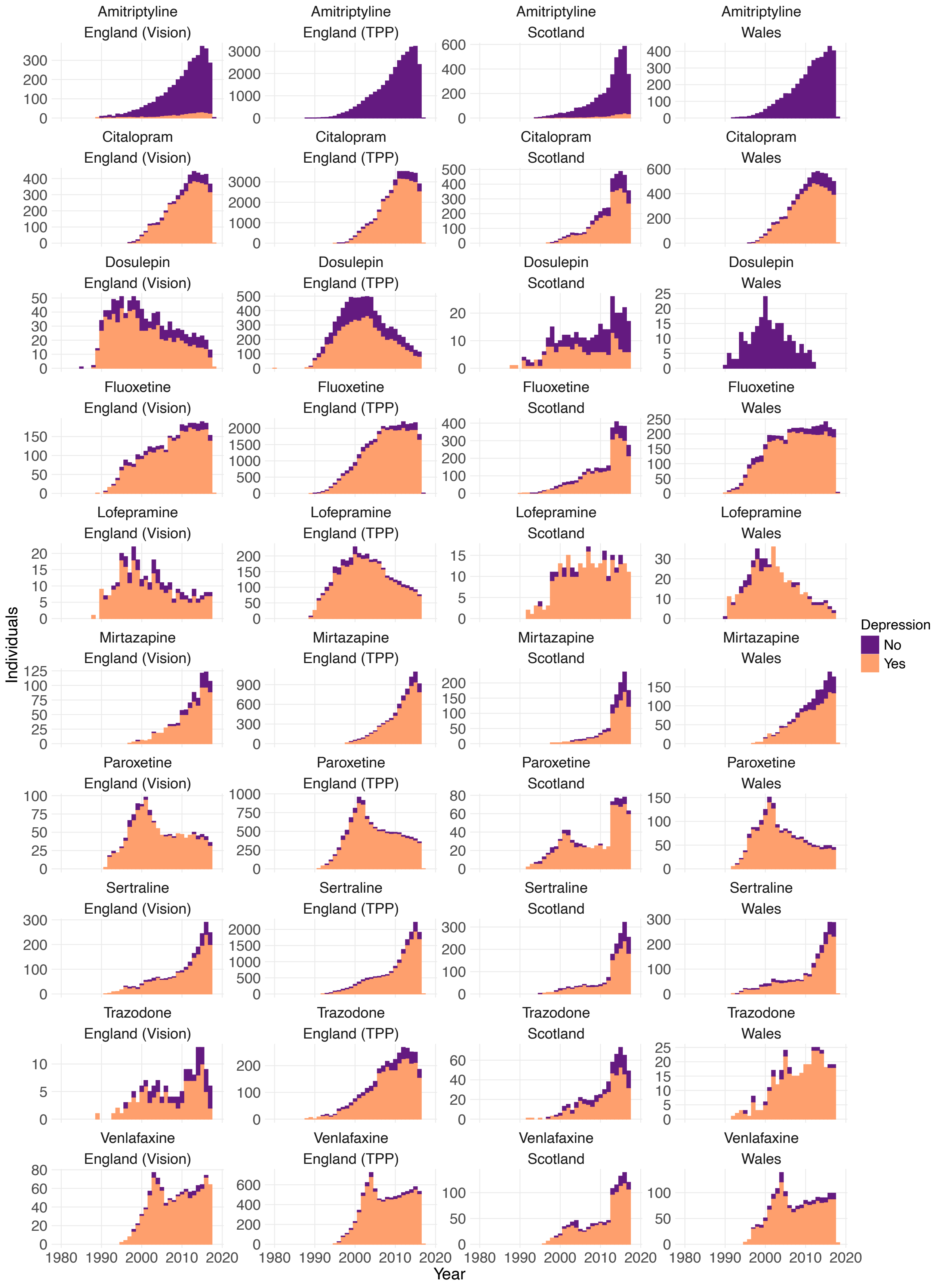

Supplementary Figure 5. Percentage of the most common ten antidepressants at the initial prescription from 1996 to 2018 across data providers

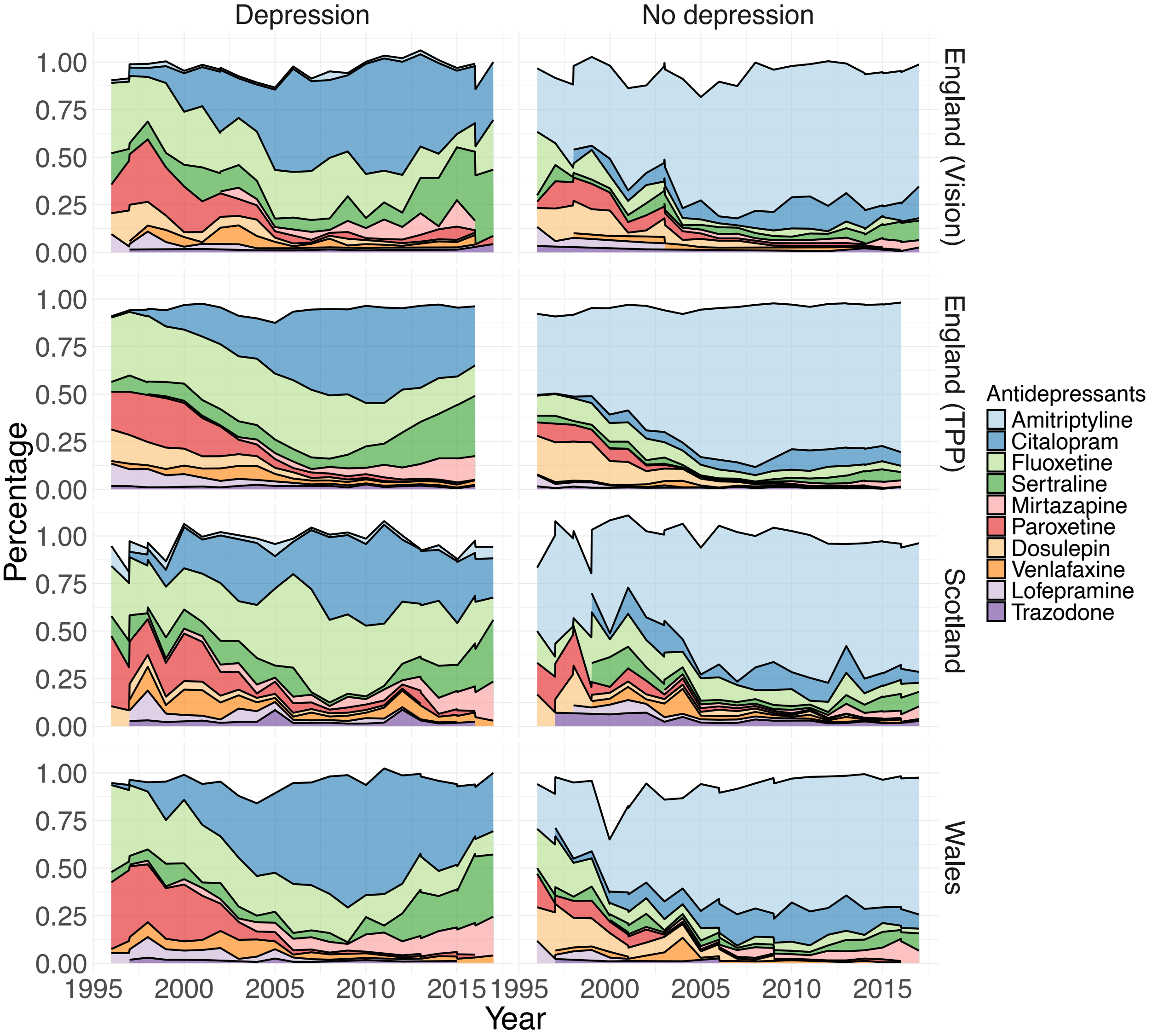

Supplementary Figure 6. Total number of antidepressant changes in depression and non-depression groups

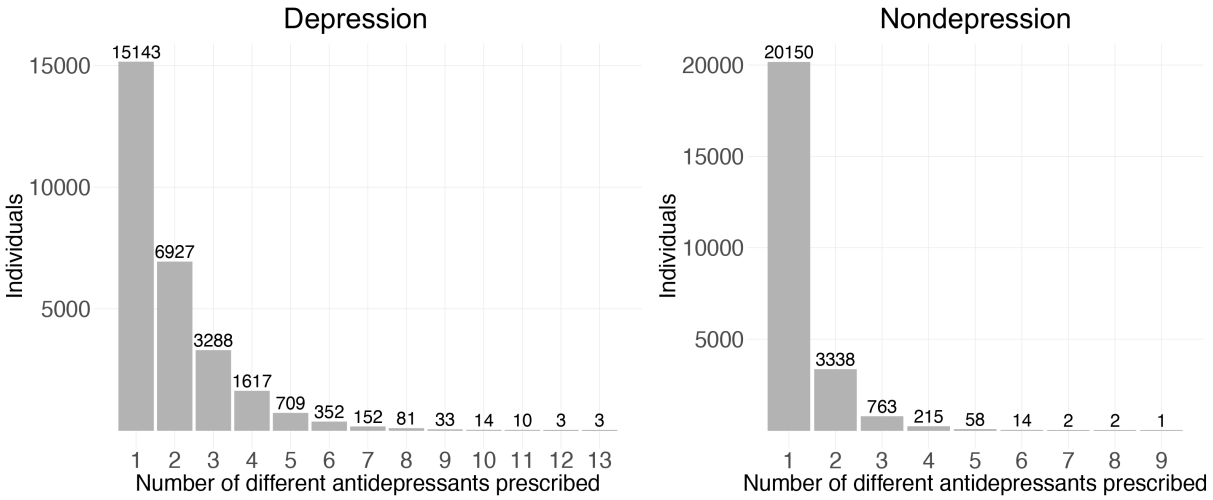

Supplementary Figure 7. Antidepressant change pathways starting from citalopram in the depression group

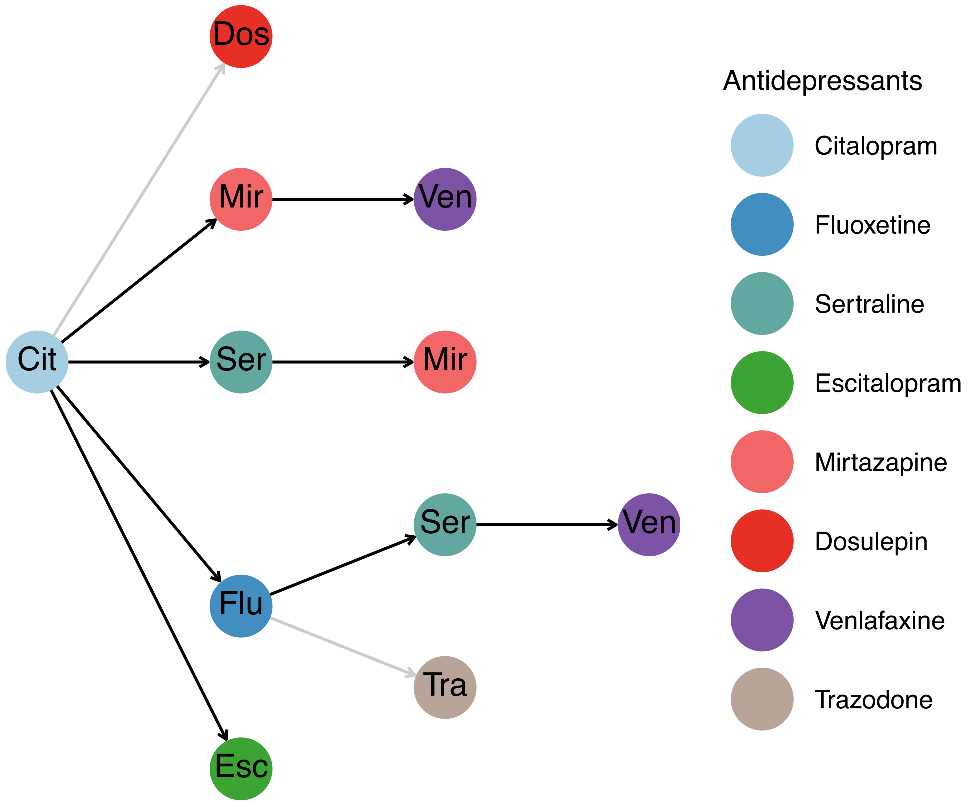

All drug pairs shown in the figure had the sample size > 10, OR > 1, and association results with the nominal significant level of $P < 0.05$, with the grey line showing unadjusted $P < 0.05$, and the black line of FDR adjusted $P < 0.05$

Supplementary Figure 8. Antidepressant change pathways starting from fluoxetine in the depression group

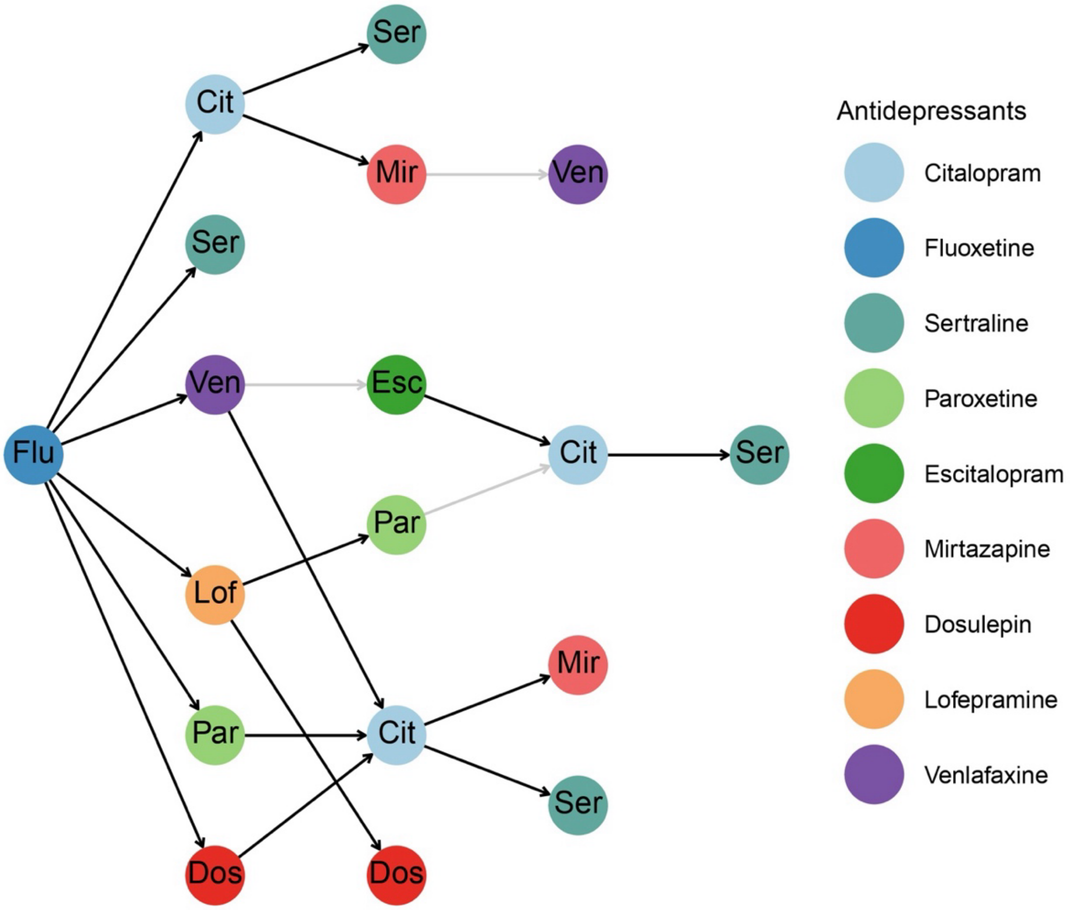

All drug pairs shown in the figure had the sample size > 10, OR > 1, and association results with the nominal significant level of P < 0.05, with the grey line showing unadjusted P < 0.05, and the black line of FDR adjusted P < 0.05

Supplementary Figure 9. Antidepressant change pathways starting from amitriptyline in the non-depression group

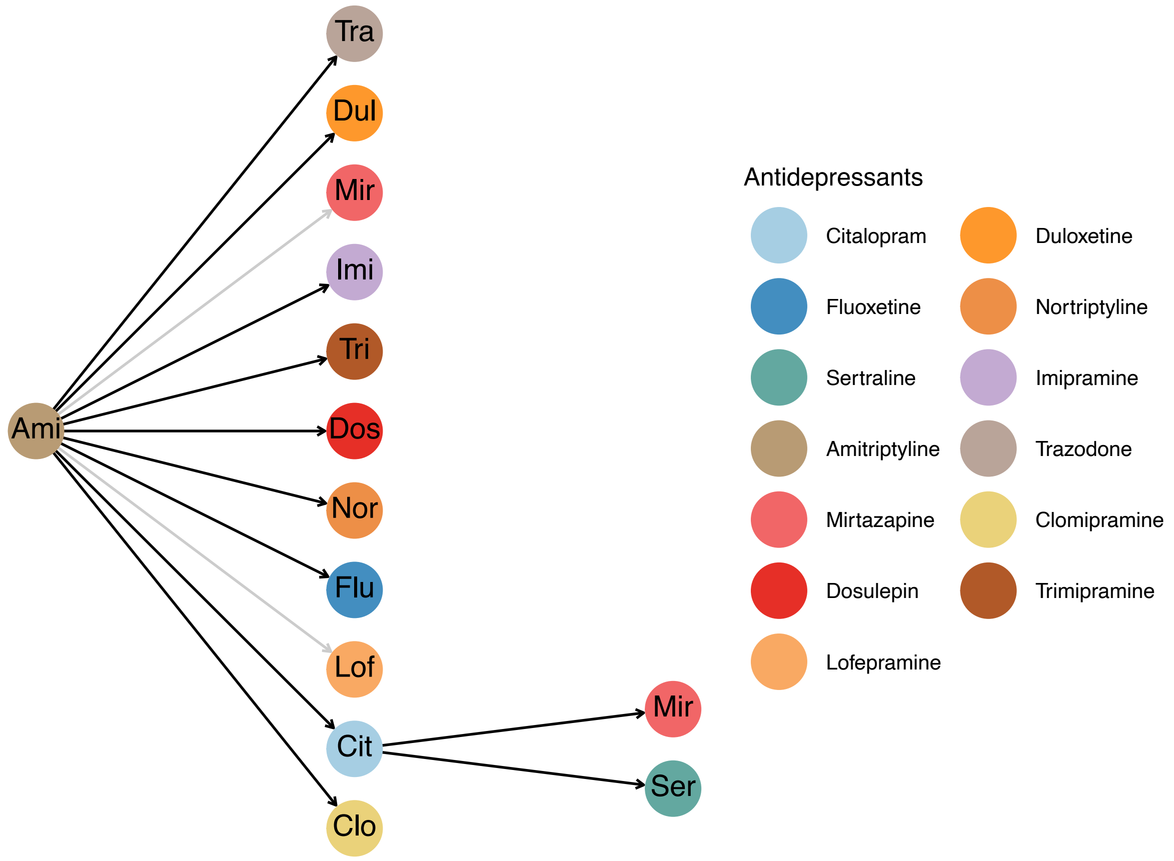

All drug pairs shown in the figure had the sample size > 10, OR > 1, and association results with the nominal significant level of $P < 0.05$, with the grey line showing unadjusted $P < 0.05$, and the black line of FDR adjusted $P < 0.05$

Supplementary Figure 10. Percentage of significant drug pairs changed across years

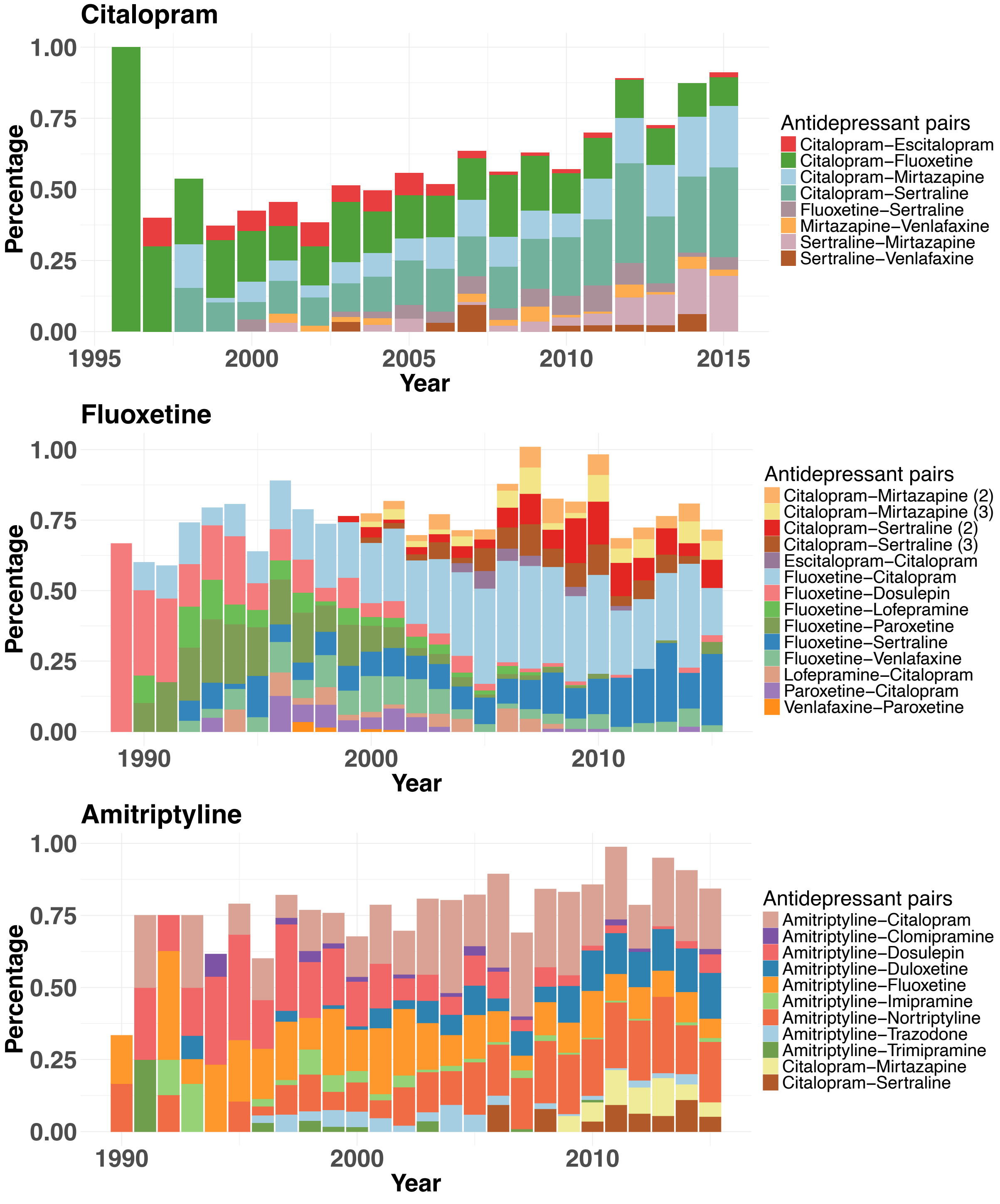

All antidepressant pairs shown in the figure reached statistical significance after multiple corrections. In fluoxetine, the number after the drug pair of citalopram-mirtazapine and citalopram-sertraline showed the order of the drug pair appeared in the antidepressant pathways, where ‘2’ indicated the drug pair was the second and third antidepressant, and ‘3’ represented the drug pair was the third and fourth antidepressant.

Supplementary Figure 11. Early and late discontinuation rates at the first ten prescribed antidepressants

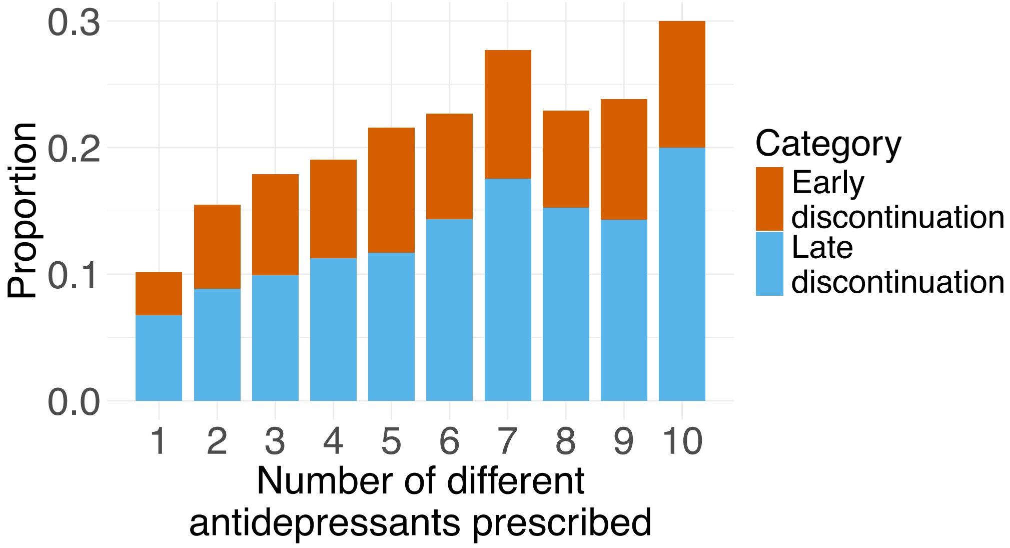

Supplementary Figure 12. Early and late discontinuation rates of each antidepressant class across number of different antidepressants prescribed

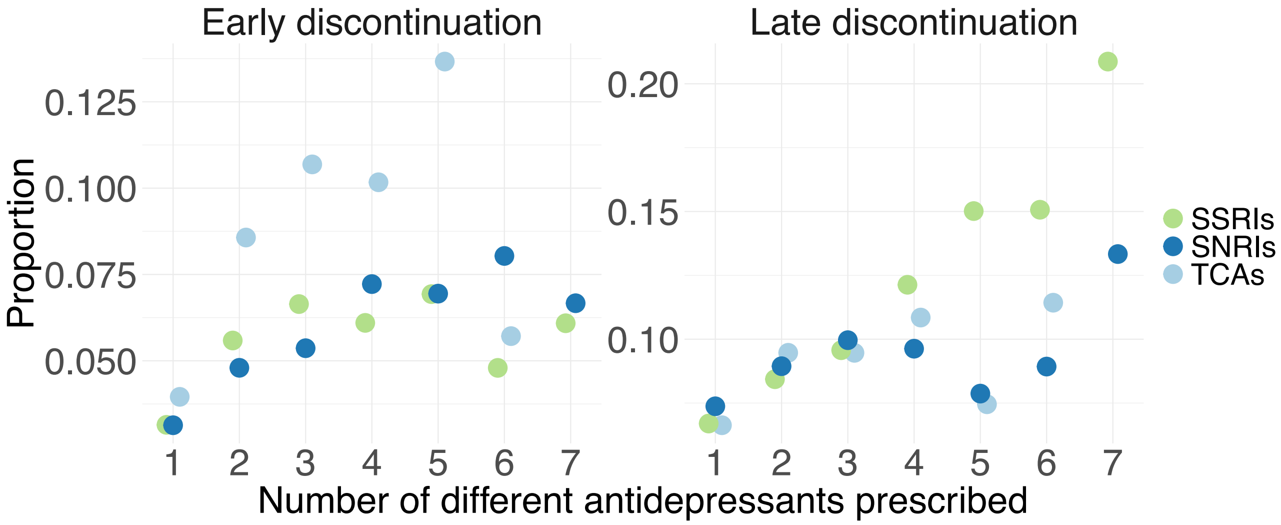

Only antidepressant class with sample size > 50 at each prescription were shown

Supplementary Figure 13. GWAS association of the number of antidepressant changes in all population

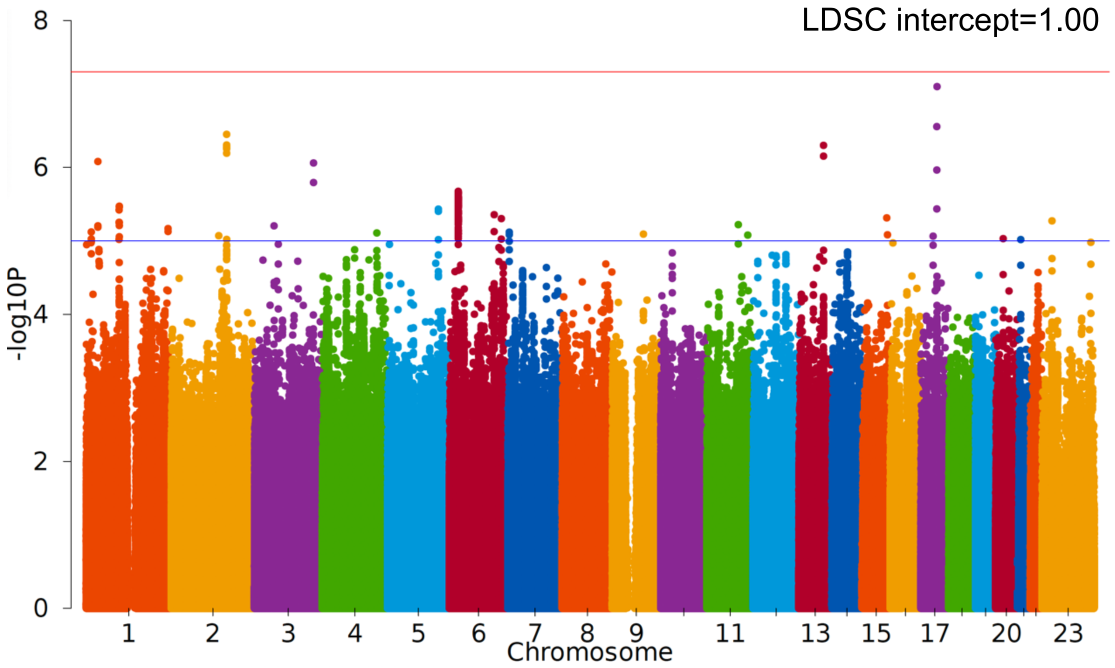

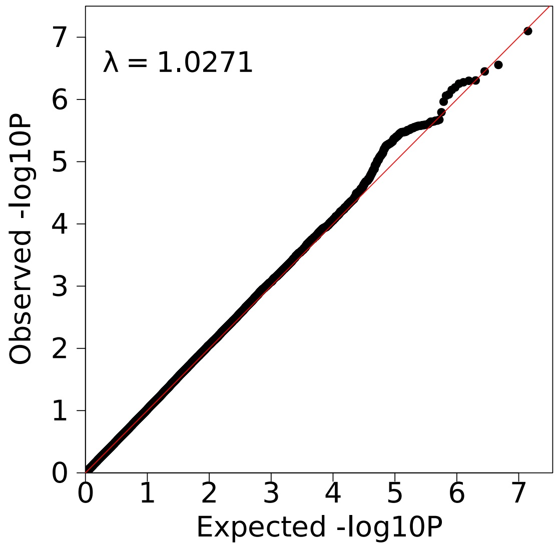

Supplementary Figure 14. GWAS association of the number of antidepressant changes in the depression group

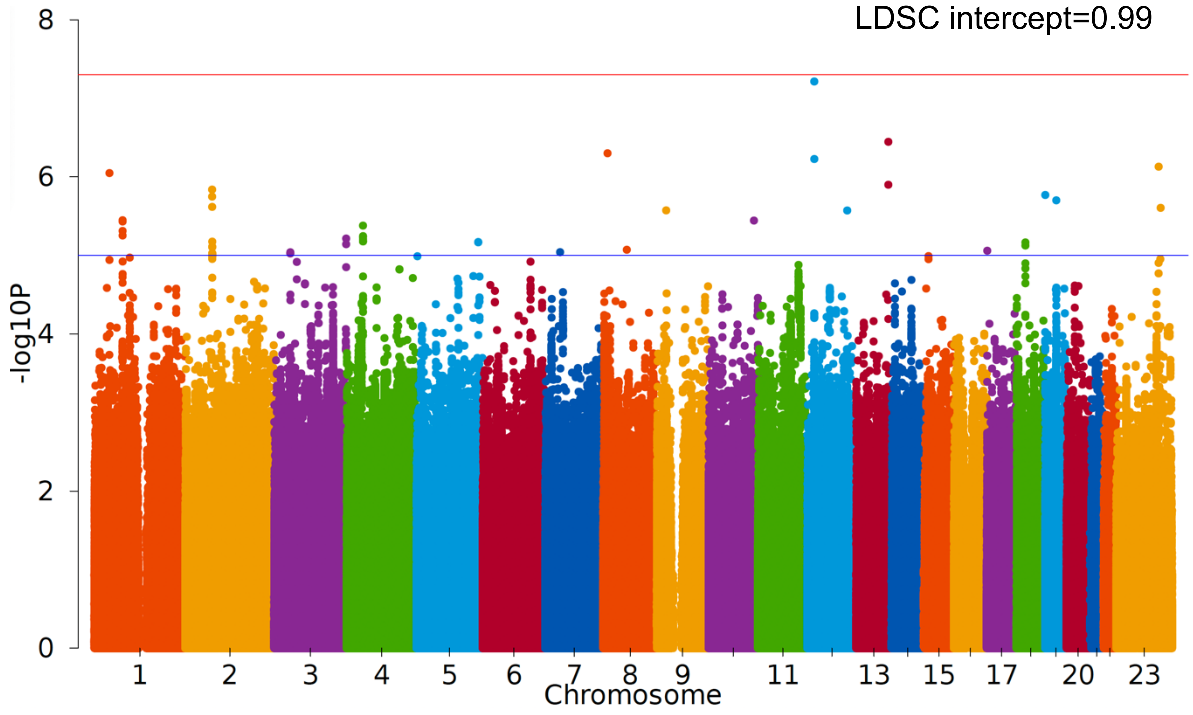

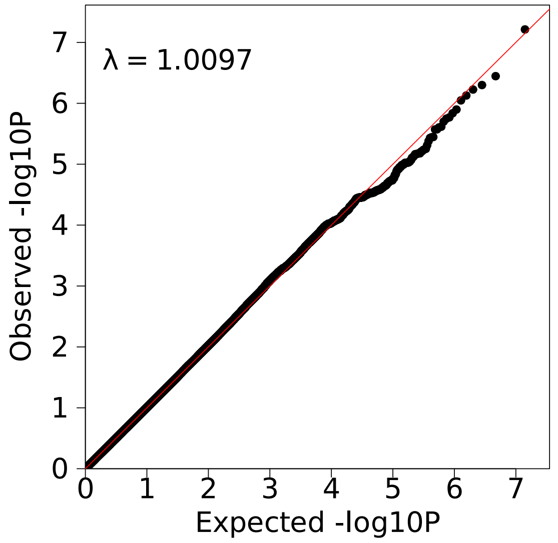

Supplementary Figure 15. GWAS association of the number of antidepressant changes in the non-depression group

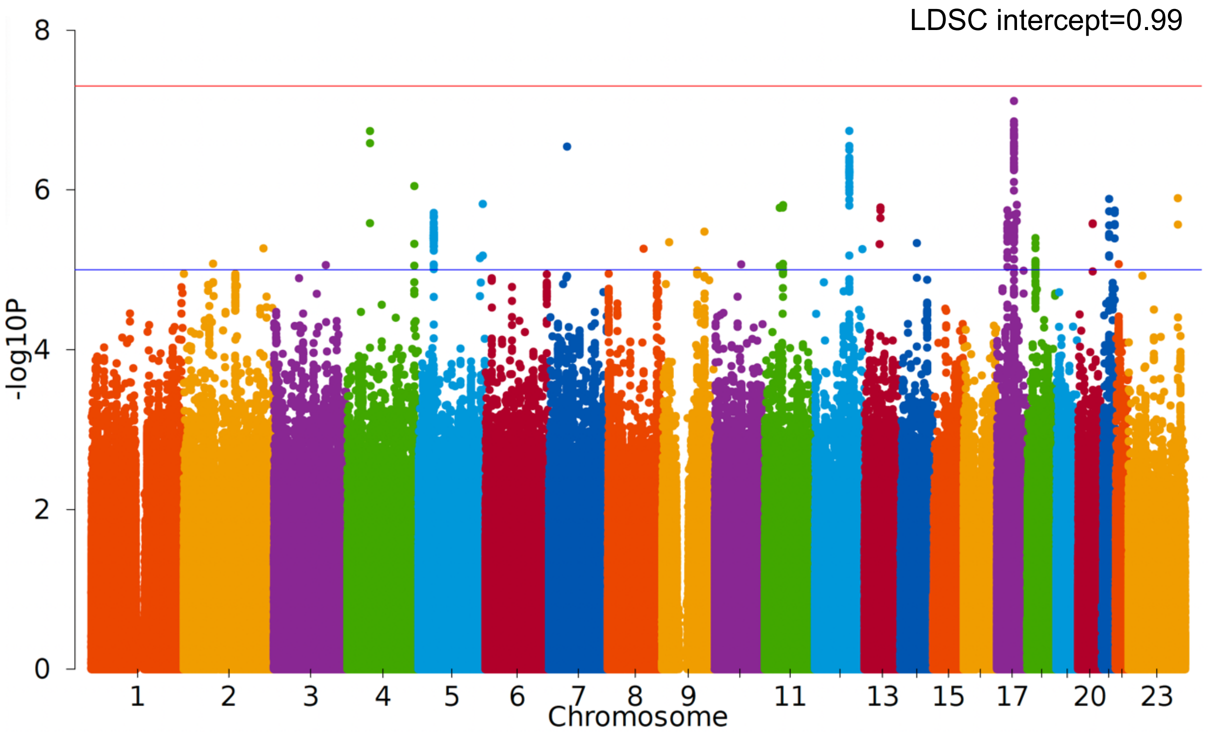

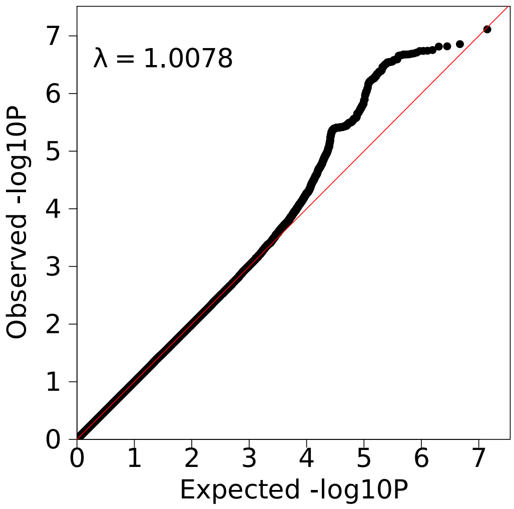

Supplementary Figure 16. GWAS association of SSRI early discontinuation

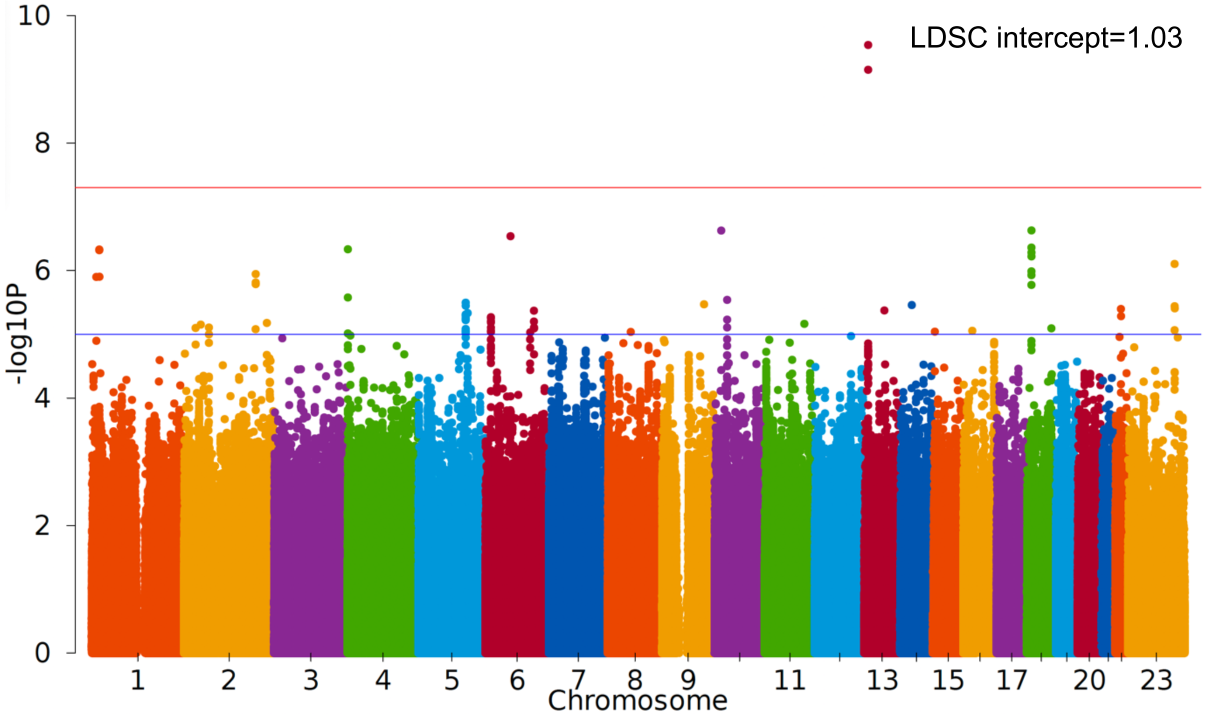

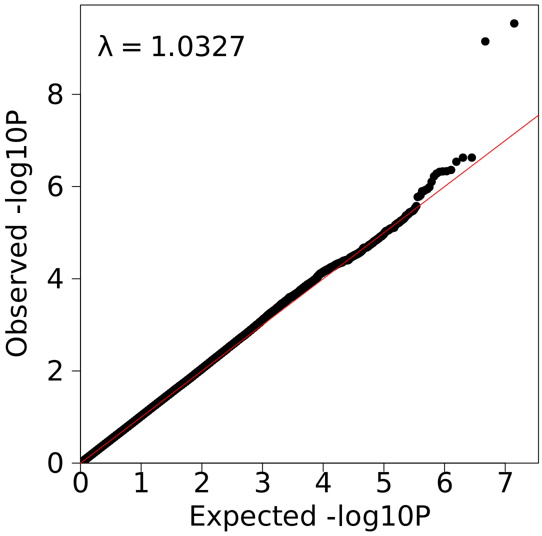

Supplementary Figure 17. GWAS association of SSRI late discontinuation

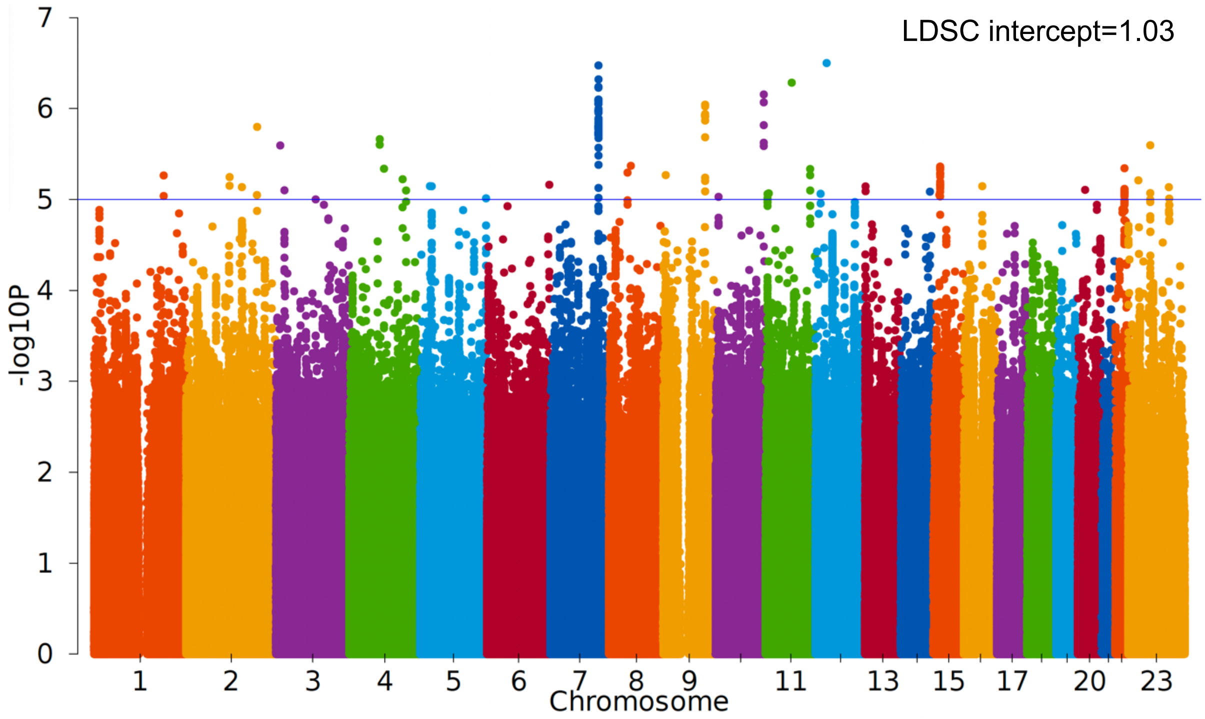

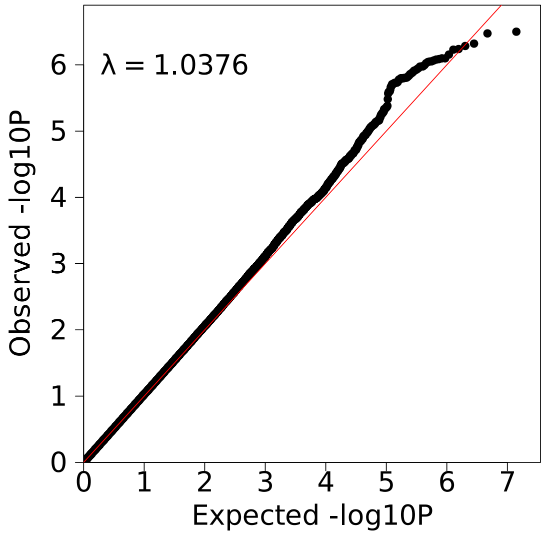

Supplementary Figure 18. Regional plot of SSRI early discontinuation

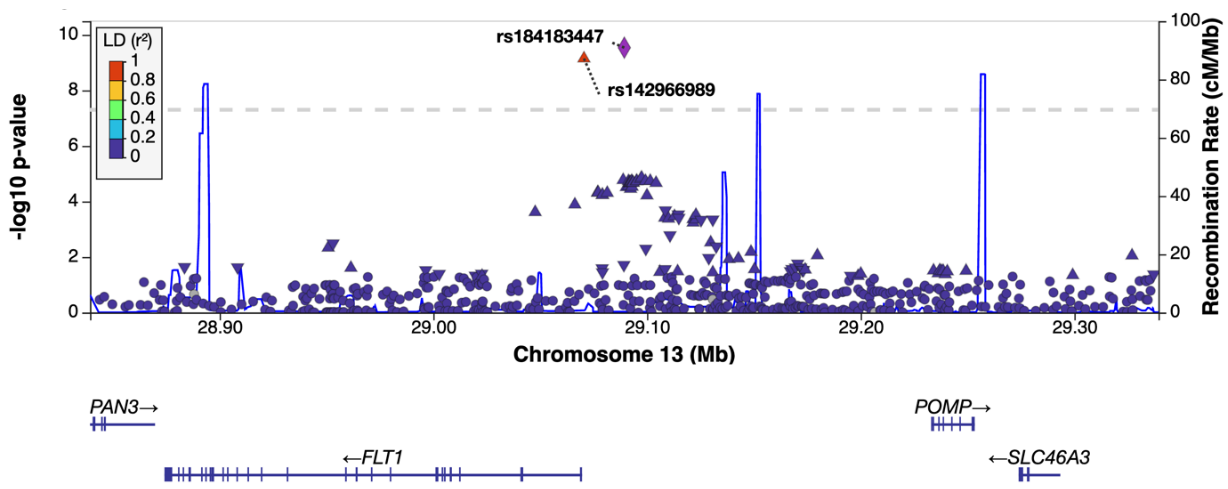

Supplementary Figure 19. SNP heritability (SNP-h2) estimated by GCTA and LDSC

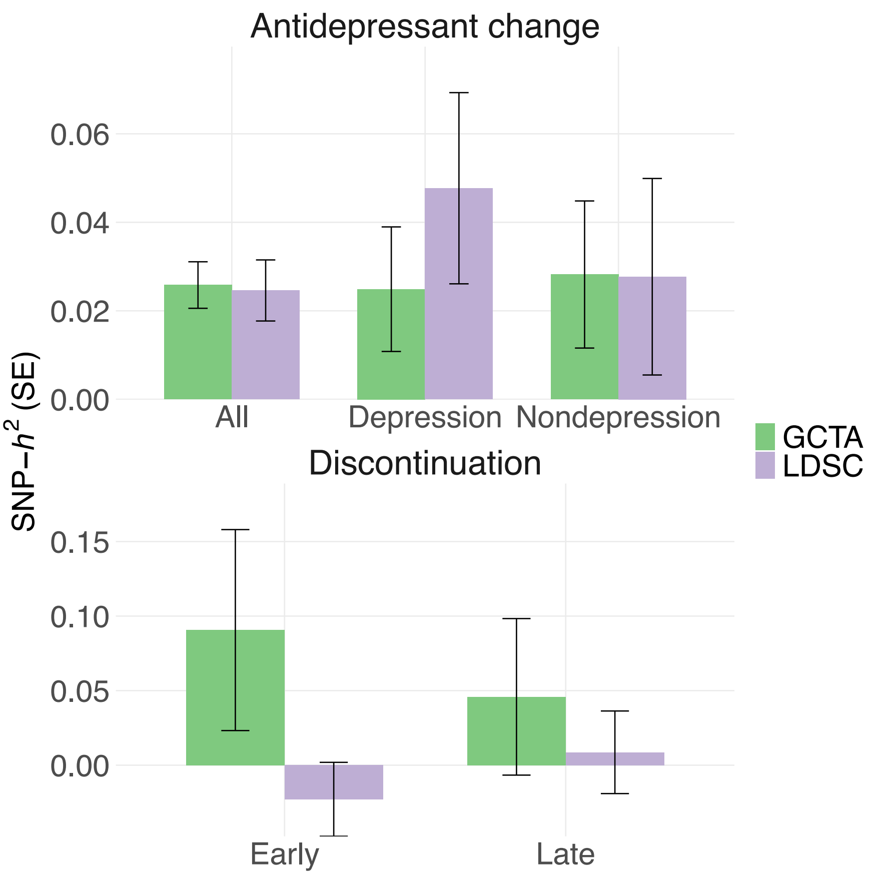

Each error bar showed standard error of the heritability estimate.

Supplementary Tables

Supplementary Table 1. Prescribed antidepressants in the UK Biobank

Drug classes	Antidepressants
SSRIs	citalopram, dapoxetine, escitalopram, fluoxetine, fluvoxamine, paroxetine, sertraline
SNRIs	duloxetine, venlafaxine
SARIs	nefazodone, trazodone
TCAs	amitriptyline, amoxapine, clomipramine, dosulepin, doxepin, imipramine, lofepramine, nortriptyline, protriptyline, trimipramine
TeCAs	maprotiline, mianserin, mirtazapine
Others	agomelatine, amitriptyline_perphenazine, bupropion, flupentixol, isocarboxazid, moclobemide, nortriptyline_fluphenazine, phenelzine, reboxetine, tranylcypromine, tranylcypromine_trifluoperazine, viloxazine, vortioxetine

Supplementary Table 2. Characteristics of individuals with antidepressant prescription in depression and non-depression groups.

	Depression		Non-depression	
	N of individuals	%	N of individuals	%
Sex				
Female	19302	68.1	14363	58.5
Male	9030	31.9	10180	41.5
Median age at first prescription	52	-	58	-
Median follow-up time (years)	12.1	-	7.9	-

Supplementary Table 3. The top ten most prescribed antidepressants in the UK Biobank

	Depression		Non-depression	
	N of individuals	% in the depression group	N of individuals	% in the non-depression group
SSRIs				
Citalopram	13228	46.7	2734	11.1
Fluoxetine	11335	40.0	2039	8.3
Sertraline	6786	24.0	1330	5.4
Paroxetine	3835	13.5	509	2.1
TCAs				
Amitriptyline	194	0.7	18426	75.1
Dosulepin	2275	8.0	1366	5.6
Lofepramine	1976	7.0	232	0.9
SNRIs				
Venlafaxine	3059	10.8	382	1.6
Others				
Mirtazapine	4038	14.3	806	3.3
Trazodone	1425	5.0	360	1.5

Supplementary Table 4. The top ten most prescribed antidepressants at the first five prescriptions

	First		Second		Third		Fourth		Fifth	
	**N**	%	**N**	**%**	**N**	%	**N**	**%**	**N**	**%**
**Depression**	28332		13189		6262		2974		1357	
Amitriptyline	76	0.27	63	0.48	24	0.38	13	0.44	9	0.66
Citalopram	7945	28.04	3150	23.88	1301	20.78	534	17.96	168	12.38
Fluoxetine	7944	28.04	2194	16.64	746	11.91	267	8.98	128	9.43
Sertraline	2902	10.24	1902	14.42	1057	16.88	513	17.25	217	15.99
Mirtazapine	1061	3.74	1283	9.73	841	13.43	426	14.32	236	17.39
Paroxetine	2529	8.93	899	6.82	233	3.72	98	3.30	43	3.17
Dosulepin	1542	5.44	480	3.64	167	2.67	56	1.88	16	1.18
Venlafaxine	937	3.31	977	7.41	581	9.28	325	10.93	136	10.02
Lofepramine	1074	3.79	488	3.70	210	3.35	112	3.77	57	4.20
Trazodone	432	1.52	406	3.08	262	4.18	159	5.35	85	6.26
**Non-depression**	24543		4393		1055		292		77	
Amitriptyline	16729	68.16	1420	32.32	217	20.57	52	17.81	8	10.39
Citalopram	1868	7.61	637	14.50	167	15.83	47	16.10	13	16.88
Fluoxetine	1471	5.99	435	9.90	98	9.29	29	9.93	5	6.49
Sertraline	797	3.25	362	8.24	122	11.56	30	10.27	15	19.48
Mirtazapine	392	1.60	269	6.12	100	9.48	31	10.62	12	15.58
Paroxetine	388	1.58	83	1.89	29	2.75	7	2.40	0	0.00
Dosulepin	1082	4.41	235	5.35	37	3.51	11	3.77	1	1.30
Venlafaxine	217	0.88	100	2.28	38	3.60	17	5.82	5	6.49
Lofepramine	146	0.59	58	1.32	17	1.61	7	2.40	2	2.60
Trazodone	221	0.90	96	2.19	34	3.22	4	1.37	2	2.60

Supplementary Table 5. Percentage of co-prescription between lithium and antidepressants

Number of different antidepressants prescribed	N of total individuals	N of lithium co-prescription	% of lithium co-prescription
1	15143	143	0.94
2	6927	114	1.6
3	3288	85	2.6
4	1617	61	3.8
5	709	45	6.3
6	352	22	6.3
7	152	16	10.5
8	81	10	12.3

Supplementary Table 7. Early and late discontinuation rates across antidepressant classes in individuals with depression

	Early discontinuation (side effects)		Late discontinuation (nonresponse)	
	N	% of total individuals	N	% of total individuals
All individuals with depression				
Total	2495	8.8	3339	11.8
SSRIs	1496	5.9	2494	9.9
SNRIs	195	5.2	347	9.2
TCAs	384	7.3	467	8.8
Excluding individuals with lithium augmentation				
Total	2446	8.7	3232	11.5
SSRIs	1479	5.9	2437	9.7
SNRIs	188	5.2	318	8.8
TCAs	370	7.2	430	8.4

Supplementary Table 9. GWAS sample size of number of antidepressant changes and discontinuation outcomes

Outcomes	N	Have discontinuation	No discontinuation	Discontinuation prevalence
Number of antidepressant changes				
All	75421			
Depression	26040			
Non-depression	22049			
SSRI discontinuation in the depression group				
Early	19178	1372	17806	7.2%
Late	17256	2315	14941	13.4%

Supplementary Table 10. Genetic correlation among antidepressant changes and discontinuation phenotypes

Phenotype 1	Phenotype 2	rg	se	P
Antidepressant change depression	Antidepressant change non-depression	0.37	0.93	0.69
Early discontinuation	Late discontinuation	0.042	0.20	0.83
Early discontinuation	Antidepressant change depression	0.058	0.35	0.87
Early discontinuation	Antidepressant change non-depression	-0.13	0.46	0.78
Late discontinuation	Antidepressant change depression	0.56	0.33	0.086
Late discontinuation	Antidepressant change non-depression	0.64	0.59	0.28

Supplementary Table 12. Chi-squared tests of discontinuation outcomes with two genetic variants in the GLAD and UK Biobank

	rs142966989 Ref: C, Alt: T			rs184183447 Ref: G, Alt: A		
	CC	CT/TT		GG	GA/AA	
UK Biobank						
Had discontinuation	1313	54	χ^2=28.737 p=8.29e-08	1311	57	χ^2=30.387 p-value=3.54e-08
No discontinuation	17433	323		17422	341	
GLAD						
Had discontinuation	1184	19	χ^2= 0.431 p-value=0.51	1184	20	χ^2= 0.226 p-value=0.63
No discontinuation	2517	50		2519	50	

Supplementary Methods

Antidepressant prescription for individuals with or without depression

In the UK Biobank, all primary care prescriptions were classified by a number of coding terms including Read v2, British National Formulary (BNF), and Dictionary of Medicines and Devices (dm+d) [1], collected from four primary care computer system suppliers: England TTP, England Vision, Wales EMIS/Vision, and Scotland EMIS/Vision. Each prescription record was annotated with the corresponding medication chemical name (e.g. citalopram, fluoxetine), and drug class (e.g. Selective serotonin reuptake inhibitors (SSRIs), Tricyclic antidepressants), following the table available at: https://doi.org/10.1101/2020.08.24.20178715. In total, 37 antidepressants were included in this study (Supplementary Table 1). For clinical events or prescription dates that preceded or same with a participant's date/year of birth, or were recorded as future dates, UK Biobank assigned predefined placeholder values (01/01/1901, 02/02/1902, 03/03/1903 and 07/07/2037), and these values were treated as missing in our analyses. More detailed description of the primary care prescription records can be found in Fabbri et al [1].

In this study, individuals prescribed antidepressants with depression were defined as those who had at least one primary care diagnosis of depressive disorders and no diagnoses for any psychotic disorders, bipolar disorder, or substance use disorders in their primary care records [1]. These diagnoses were classified by the clinical coding terms version 2 (Read v2) and version 3 (CTV3 or Read v3), which have been used in UK primary care since 1985. Following the study from Fabbri et al. [1], both Read v2 and Read v3 clinical codes were classified into diagnostic groups such as depressive disorders, bipolar disorders, psychosis, and substance use disorders, and the Read v2 clinical codes were mapped to the corresponding Read v3 clinical codes (https://doi.org/10.1101/2020.08.24.20178715).

For individuals prescribed antidepressants without depression, they were included if they had at least one antidepressant prescription but without any depression measures identified in the UK Biobank. This included:

1) Primary care diagnoses of depressive disorders as described above [1].

2) Broad depression: self-reported help-seeking behaviour for mental health difficulties, defined as Individual answering 'yes' to either of the following questions: 'Have you ever seen a general practitioner (GP) for nerves, anxiety, tension, or depression?' (data field 2090) or 'Have you ever seen a psychiatrist for nerves, anxiety, tension or depression?' (data field 2100) [2].

3) Lifetime depression: defined using the Composite International Diagnostic Interview Short Form (CIDI-SF) that was part of the second Mental Health Questionnaire (MHQ) [3]. Criteria for lifetime major depressive episode were in accordance with the DSM-V.

4) Self-reported depression: past and current depression self-reported to the interviewer (a trained nurse) (data field 20002), or answering 'yes' to the question 'During the past six months have you had depression?' (data field 120044).

5) Hospital inpatient diagnosis: based on 3-digit ICD10 codes for F32 Depressive episode, F33 Recurrent depressive disorder, F34 Persistent mood [affective] disorders, F38 Other mood [affective] disorders, F39 Unspecified mood [affective] disorder, from both primary (data field 41202) and secondary diagnoses (data field 41204) [2].

There were 82,633 individuals who were prescribed at least one recorded antidepressant prescription. Among them, 28,332 individuals had at least one primary care diagnosis of depression, and 24,543 individuals did not have any depression identified from the above measures. The remaining 29,758 individuals did not meet the criteria for either group, so they were excluded for the subgroup analyses.

Augmentation of lithium prescription

We extract lithium prescriptions from a list of Read v2, BNF, and dm+d coding terms based on the previous study from the UK Biobank [4]. Among the 28,332 individuals in the depression group, 283 had been prescribed lithium, of whom 234 (0.83%) had lithium co-prescribed with at least one antidepressant. The detailed number of lithium co-prescription with the number of changed antidepressants was presented in the Supplementary Table 5.

Antidepressant discontinuation likely due to side effects or nonresponse in individuals with depression

We identified two discontinuation phenotypes of SSRIs in the depression group, where early discontinuation was likely due to side effects and late discontinuation was likely due to nonresponse. For early discontinuation, controls were defined as individuals prescribed any SSRIs more than six weeks and at least three times in a depression episode to ensure they do not suffer from side effects. For late discontinuation, controls were restricted to individuals prescribed any SSRIs for at least six weeks and more than three times in a depression episode, and they either switched to another antidepressant with a break longer than 90 days or had no switch to another drug afterwards. This is to ensure individuals received adequate antidepressant treatment duration in a depression episode without changing to another antidepressant likely due to nonresponse. Individuals who experienced any early or late discontinuation of SSRI treatment were excluded from their respective control groups.

Genotype and imputation

Genome-wide genotyping of all UK Biobank participants was performed using two overlapping arrays, containing ~800,000 markers. Quality control of autosomal genotype data was conducted to correct for potential confounding factors such as genotyping array effects, batch effects, and deviations from Hardy–Weinberg equilibrium (HWE) [5]. SNPs were excluded if they showed high level of missingness (>

2%) or significant deviation from HWE ($p < 1 \times 10^{-8}$). Individuals were removed if they exhibited abnormal heterozygosity (as defined in centralized QC), excessive genotype missingness (> 5%), third-degree or closer relatedness (KING r < 0.044), or discordance between genetic sex and self-reported gender. Population structure was evaluated using principal component analysis (PCA), and individuals of European ancestry were identified using four-means clustering based on the first two genetic principal components. Genotype imputation was performed using the Haplotype Reference Consortium (HRC) and UK10K reference panels [6]. Quality control was performed on the imputed genotype, restricting analyses to variants with imputation quality INFO score < 0.4, minor allele frequency > 0.01, missing rate < 0.05, and Hardy-Weinberg equilibrium exact test p-value > 1×10^{-8}.

Heritability estimate and genetic correlation

We applied both Genome-wide Complex Trait Analysis (GCTA) and linkage disequilibrium score regression (LDSC) [7,8] to evaluate SNP-based heritability. For binary phenotypes, heritability estimates were transformed to the liability scale using the prevalence observed in the dataset. GCTA models were adjusted for batch, array, the first ten genetic principal components, data provider, birth year, and year of the first prescription. Genetic correlations with 16 selected psychiatric and physical traits were computed using LDSC, constraining the regression intercept to 1 [9–22]. The FDR method was applied for multiple correction across all genetic correlation tests.

Polygenic score

Polygenic scores (PGSs) were calculated using SBayesR, which is a Bayesian regression method to estimate SNP effect sizes while accounting for linkage disequilibrium and polygenic architecture [23]. We used a shrunk sparse matrix as the reference LD panel described in Lloyd-Jones et al [23]. Since all SNPs included in each summary statistic had passed quality control in the original GWAS, no additional filtering was applied before PGS construction [9,11,17,20]. The number of SNPs included in the PGS for MDD, schizophrenia, bipolar disorder, and ADHD were 1,025,837, 986,493, 1,065,893, and 999,535, respectively. For continuous phenotypes like antidepressant change, a Gamma regression was applied to assess the explained variance of PRS associations. For binary phenotypes including early and late SSRI discontinuation, logistic regression was applied, with Nagelkerke's R2 used to evaluate the explained variance. The population prevalence was assumed same to the dataset. All models were adjusted for the first ten ancestry-specific principal components, data provider, birth year, year of the first prescription and genotyping batch. FDR correction was applied across all PGS tests.

External replication of significant genetic variants

We evaluated the two genome-wide significant variants identified in the SSRI early discontinuation in an independent UK-based population cohort - the Genetic Links to Anxiety and Depression (GLAD) study, which is the world's largest single study of depression and anxiety[24]. In GLAD, individuals from the UK were recruited via

traditional and social media, as well as clinic-based recruitment, meeting DSM-5 criteria for major depressive disorder (MDD) or any anxiety disorders. Each participant provided self-reported side effects for commonly prescribed antidepressants in the UK, including six SSRIs (citalopram, fluoxetine, sertraline, paroxetine, escitalopram, fluvoxamine). Discontinuation due to side effects was indicated by the response to whether the participant had to stop taking the antidepressant due to side effects. We used self-reported discontinuation at the first SSRI as the outcome to approximate the early discontinuation phenotype derived from primary care records in the UK Biobank.

We identified a total of 6,078 individuals answered this question, and the rate of discontinuation due to side effect was 32%. Of these, 5,029 participants had genotype data available. We examined the distribution of the two significant variants identified from the UK Biobank (rs142966989, rs184183447) for the discontinuation due to side effects and tested their statistical significance using a chi-squared test. As shown in Supplementary Table 12, neither variant showed significant effect on the discontinuation due to side effects in the GLAD cohort.

Reference

1. Fabbri, C. *et al.* Genetic and clinical characteristics of treatment-resistant depression using primary care records in two UK cohorts. *Molecular Psychiatry* **26**, 3363–3373 (2021).
2. Howard, D. M. *et al.* Genome-wide association study of depression phenotypes in UK Biobank identifies variants in excitatory synaptic pathways. *Nat Commun* **9**, 1470 (2018).
3. Davis, K. A. S. *et al.* The UK Biobank mental health enhancement 2022: Methods and results. *PLoS One* **20**, e0324189 (2025).
4. Mutz, J. *et al.* The duration of lithium use and biological ageing: telomere length, frailty, metabolomic age and all-cause mortality. *GeroScience* **46**, 5981–5994 (2024).
5. Bycroft, C. *et al.* The UK Biobank resource with deep phenotyping and genomic data. *Nature* **562**, 203–209 (2018).
6. The UK10K Consortium *et al.* The UK10K project identifies rare variants in health and disease. *Nature* **526**, 82–90 (2015).
7. Schizophrenia Working Group of the Psychiatric Genomics Consortium *et al.* LD Score regression distinguishes confounding from polygenicity in genome-wide association studies. *Nat Genet* **47**, 291–295 (2015).
8. Yang, J., Lee, S. H., Goddard, M. E. & Visscher, P. M. GCTA: A Tool for Genome-wide Complex Trait Analysis. *The American Journal of Human Genetics* **88**, 76–82 (2011).
9. Demontis, D. *et al.* Genome-wide analyses of ADHD identify 27 risk loci, refine the genetic architecture and implicate several cognitive domains. *Nat Genet* **55**, 198–208 (2023).
10. Friligkou, E. *et al.* Gene discovery and biological insights into anxiety disorders from a large-scale multi-ancestry genome-wide association study. *Nat Genet* **56**, 2036–2045 (2024).
11. O’Connell, K. S. *et al.* Genomics yields biological and phenotypic insights into bipolar disorder. *Nature* **639**, 968–975 (2025).
12. Johnston, K. J. A. *et al.* Genome-wide association study of multisite chronic pain in UK Biobank. *PLoS Genet* **15**, e1008164 (2019).
13. Saunders, G. R. B. *et al.* Genetic diversity fuels gene discovery for tobacco and alcohol use. *Nature* **612**, 720–724 (2022).
14. International Headache Genetics Consortium *et al.* Meta-analysis of 375,000 individuals identifies 38 susceptibility loci for migraine. *Nat Genet* **48**, 856–866 (2016).

15. Watanabe, K. *et al.* Genome-wide meta-analysis of insomnia prioritizes genes associated with metabolic and psychiatric pathways. *Nat Genet* **54**, 1125–1132 (2022).
16. 23andMe Research Team *et al.* Gene discovery and polygenic prediction from a genome-wide association study of educational attainment in 1.1 million individuals. *Nat Genet* **50**, 1112–1121 (2018).
17. Trubetskoy, V. *et al.* Mapping genomic loci implicates genes and synaptic biology in schizophrenia. *Nature* **604**, 502–508 (2022).
18. Verma, A. *et al.* Diversity and scale: Genetic architecture of 2068 traits in the VA Million Veteran Program. *Science* **385**, eadj1182 (2024).
19. Eijsbouts, C. *et al.* Genome-wide analysis of 53,400 people with irritable bowel syndrome highlights shared genetic pathways with mood and anxiety disorders. *Nat Genet* **53**, 1543–1552 (2021).
20. Adams, M. J. *et al.* Trans-ancestry genome-wide study of depression identifies 697 associations implicating cell types and pharmacotherapies. *Cell* **188**, 640-652.E9 (2025).
21. 23andMe Research Team *et al.* Meta-analysis of genome-wide association studies for neuroticism in 449,484 individuals identifies novel genetic loci and pathways. *Nat Genet* **50**, 920–927 (2018).
22. Berndt, S. I. *et al.* Genome-wide meta-analysis identifies 11 new loci for anthropometric traits and provides insights into genetic architecture. *Nat Genet* **45**, 501–512 (2013).
23. Lloyd-Jones, L. R. *et al.* Improved polygenic prediction by Bayesian multiple regression on summary statistics. *Nat Commun* **10**, 5086 (2019).
24. Davies, M. R. *et al.* The Genetic Links to Anxiety and Depression (GLAD) Study: Online recruitment into the largest recontactable study of depression and anxiety. *Behaviour Research and Therapy* **123**, 103503 (2019).
